# CBS-derived H_2_S facilitates host colonization of *Vibrio cholerae* by promoting the iron-dependent catalase activity of KatB

**DOI:** 10.1371/journal.ppat.1009763

**Published:** 2021-07-20

**Authors:** Yao Ma, Xiaoman Yang, Hongou Wang, Zixin Qin, Chunrong Yi, Changping Shi, Mei Luo, Guozhong Chen, Jin Yan, Xiaoyun Liu, Zhi Liu

**Affiliations:** 1 Department of Biotechnology, College of Life Science and Technology, Huazhong University of Science and Technology, Wuhan, China; 2 Department of Microbiology, School of Basic Medical Sciences, Peking University Health Science Center, Beijing, China; University of Toronto, CANADA

## Abstract

Sensing and resisting oxidative stress is critical for *Vibrio cholerae* to survive in either the aquatic environment or the gastrointestinal tract. Previous studies mainly focused on the mechanisms of oxidative stress response regulation that rely on enzymatic antioxidant systems, while functions of non-enzymatic antioxidants are rarely discussed in *V*. *cholerae*. For the first time, we investigated the role of hydrogen sulfide (H_2_S), the simplest thiol compound, in protecting *V*. *cholerae* against oxidative stress. We found that degradation of L-cysteine by putative cystathionine β-synthase (CBS) is the major source of endogenous H_2_S in *V*. *cholerae*. Our results indicate that intracellular H_2_S level has a positive correlation with *cbs* expression, while the enhanced H_2_S production can render *V*. *cholerae* cells less susceptible to H_2_O_2_ *in vitro*. Using proteome analysis and real-time qPCR assay, we found that *cbs* expression could stimulate the expression of several enzymatic antioxidants, including reactive oxygen species (ROS) detoxifying enzymes SodB, KatG and AhpC, the DNA protective protein DPS and the protein redox regulator Trx1. Assays of ROS detoxification capacities revealed that CBS-derived H_2_S could promote catalase activity at the post-translational level, especially for KatB, which serves as an important way that endogenous H_2_S participates in H_2_O_2_ detoxification. The enhancement of catalase activity by H_2_S is achieved through facilitating the uptake of iron. Adult mice experiments showed that *cbs* mutant has colonization defect, while either complementation of *cbs* or exogenous supplement of N-Acetyl-L-Cysteine restores its fitness in the host environment. Herein, we proposed that *V*. *cholerae* regulates CBS-dependent H_2_S production for better survival and proliferation under ROS stress.

## Author summary

*Vibrio cholerae* is the etiological agent of cholera, the diarrheal disease, according to WHO epidemiological record, has been experiencing a high number of cases over the past few years. Upon ingestion by humans, *V*. *cholerae* needs to overcome multiple stresses in the host gastrointestinal tract, including the reactive oxygen species produced by host innate immune system. Here, we resolved the source of endogenous H_2_S in *V*. *cholerae* and its physiological functions. We found that cystathionine β-synthase (CBS)-catalyzed H_2_S production from L-cysteine enhanced the resistance of *V*. *cholerae* to oxidative stress, leading to increased survival *in vitro* and promoted colonization in an adult mouse model. We identified two mechanisms for the cytoprotective effects of endogenous H_2_S: 1) stimulating the expression of enzymatic antioxidant systems and 2) promoting the activity of catalases, especially KatB, at the post-translational level. We also found that H_2_S facilitated iron uptake, which may be responsible for its indirect promotion of KatB activity. Our results broaden the understanding of the physiological functions of H_2_S in bacteria and provide a new perspective on the contribution of endogenous small molecule gases to the functional adaptability of bacteria.

## Introduction

*Vibrio cholerae* is the pathogen of cholera, the endemic diarrheal disease for which the number of cases has continued to be high over the last few years according to WHO epidemiological record. During 2019, 923,037 cases were notified from 31 countries, with most cases reported in Southern and Western Asia (93%), and the highest case-fatality rate was announced for the African Region (1.6%) [1]. To survive in an aquatic environment, *V*. *cholerae* needs to overcome the reactive oxygen species (ROS) generated through the photooxidation of dissolved organic matter which is triggered by solar radiation [2]. As an enteric pathogen, *V*. *cholerae* also withstands diverse stress conditions during host infection, including the ROS produced by gut epithelia in response to the mitochondrial activity activators released by commensal and pathogenic bacteria, and increased oxidative stress resulted from ROS-induced inflammatory response [3]. Thus, sensing and resisting oxidative stress is critical for *V*. *cholerae* to survive in the host gastrointestinal (GI) tract. In general, bacterial safeguarding capabilities against ROS mainly rely on enzymatic antioxidant systems, while non-enzymatic systems also play a role in maintaining cellular redox balance. ROS-degrading enzymes in bacteria include superoxide dismutases (SODs), catalases (CATs), thiol-based peroxidases (i.e. peroxiredoxins) and superoxide reductases (SORs) [4,5]. In *V*. *cholerae*, studies on ROS scavenging focus on the enzymatic antioxidant systems and related regulatory mechanisms. The reported enzymatic ROS scavengers include three SODs SodA (Mn- Sod), SodB (Fe-Sod) and SodC (Cu/Zn-Sod), two CATs KatB and KatG, and three thiol-based peroxidases PrxA, AhpC and OhrA [6–9].

Low-molecular-weight (LMW) thiols are common, non-enzymatic antioxidants in bacteria. The major LMW thiols that function as redox buffers include glutathione, the best-studied LMW thiol which is widespread in Gram-negative bacteria; cysteine, coenzyme A, and bacillithiol, which are utilized by *Bacillus* and *Staphylococcus* species; mycothiol, that is present in *Actinomycetes*; and ergothioneine, which is produced by mycobacteria [10]. Hydrogen sulfide (H_2_S), the simplest thiol, is primarily known as toxic gas and has recently emerged as an endogenously generated signaling molecule in mammals, plants, and bacteria [11–13]. Signaling by H_2_S is achieved predominantly via a post-translational modification of cysteine residues in proteins, called *S*-Sulfhydration or persulfidation, and generally increases the activity of target proteins [14,15]. Synthesis of endogenous H_2_S from cysteine metabolism is conserved from prokaryotes to mammals. The enzymes involved are cystathionine β-synthase (CBS) and cystathionine γ-lyase (CSE) within the transsulfuration pathway and 3-mercaptopyruvate sulfurtransferase (3MST) [16,17]. As for certain kinds of bacteria surviving in the GI tract, H_2_S can be produced via assimilatory sulfite reduction, or dissimilatory sulfate reduction that are usually observed in sulfate-reducing bacteria [18].

Endogenous H_2_S has been proved to protect bacterial cells from oxidative stress in *Escherichia coli*, *Pseudomonas aeruginosa*, *Staphylococcus aureus*, and *Bacillus anthracis* [17,19]. The proposed cytoprotective mechanisms in *E*. *coli* include: 1) homeostatic control of cysteine levels by H_2_S biosynthesis, since high levels of intracellular cysteine promote the Fenton reaction, 2) direct interaction between H_2_S and ROS or free iron, and 3) stimulation on SOD and CAT activity by H_2_S [17,19,20]. Compared to the in-depth research on H_2_S signaling in mammals, little is known about the biological function of H_2_S in bacteria. With the help of progressively improved persulfide proteomic analyses, the possible relationship between H_2_S signaling and virulence was reported in human pathogens *S*. *aureus* and *Enterococcus faecalis* [13,21]. However, the biological function of H_2_S in *V*. *cholerae* is largely unknown.

In this study, we investigated the role of endogenous H_2_S in cytoprotection against oxidative stress in *V*. *cholerae* for the first time. We found that degradation of L-cysteine is the main source of H_2_S production in *V*. *cholerae* and the ortholog of CBS is the critical enzyme involved in the related biochemical reaction. Our results indicated that intracellular H_2_S levels are positively correlated with *cbs* expression, and that the enhanced H_2_S production makes *V*. *cholerae* cells resistant to H_2_O_2_ *in vitro*. As shown in the present study, this cytoprotective effect of H_2_S is achieved by stimulating the expression of multiple enzymatic antioxidants, as well as by increasing the activity of catalases, particularly KatB, at the post-translational level. Furthermore, we found that the promotion of catalase activity is associated with facilitation of iron uptake. Most importantly, we provide evidence that CBS-derived H_2_S can improve the fitness of *V*. *cholerae* by increasing its tolerance to oxidative stress in adult mouse model.

## Results

### CBS is the key enzyme for H_2_S production in *V. cholerae*

**Fig 1:**
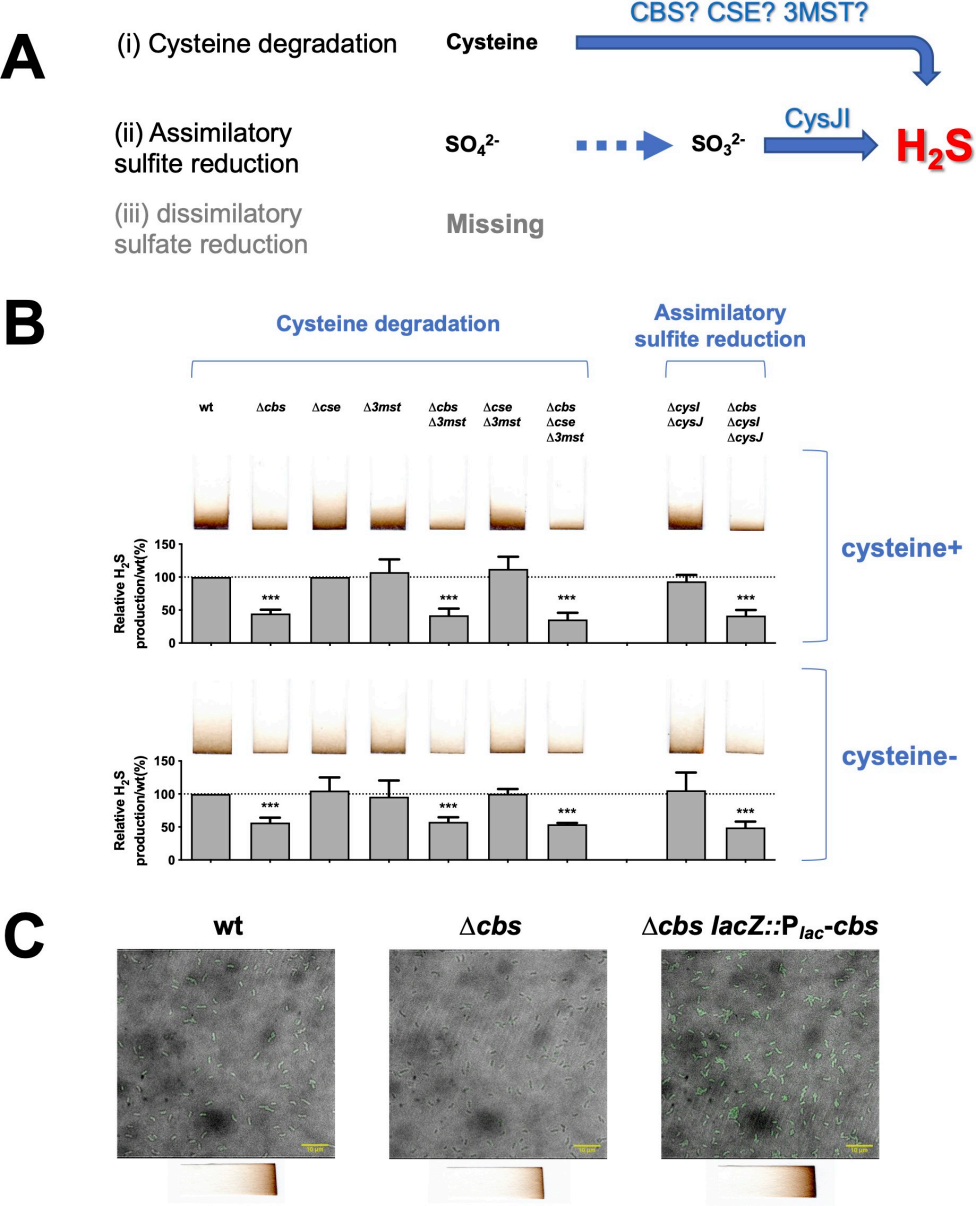
CBS is the key enzyme for H_2_S production in *V*. *cholerae*. (A). Schematic illustration of the proposed pathways for H_2_S biogenesis in *V*. *cholerae*. CBS, CSE and 3MST are key enzymes in cysteine degradation that are conserved from bacteria to mammals, while NADPH-dependent sulfite reductase (CysJI) catalyzes the last step in assimilatory sulfite reduction. (B). Cysteine degrading enzyme CBS is essential for H_2_S production. To investigate the contribution of cysteine degradation (*cbs*, *cse*, and *3mst*) and assimilatory sulfite reduction (*cysI* and *cysJ*) to H_2_S synthesis in *V*. *cholerae*, H_2_S production was detected with Pb(Ac)_2_ paper strips with (upper) or without (bottom) supplementation of 200 μM L-cysteine hydrochloride in the culture medium. Stained paper strips were scanned and quantified with ImageJ. Average H_2_S level of the wild-type (WT) was set to 100% for subsequent normalization. Three replicates were sampled for each strain. Asterisks indicate statistically significant differences compared to WT as found by *t*-test (***, *p*-value < 0.001). (C). Real-time detection of intracellular H_2_S signals. H_2_S signal was detected by WSP-5 (working concentration 15 μM, green signal) for bacteria that were cultivated in LB. Picture captured in the 488–524 channel was merged with the bright field image. Scale bar indicates length of 10 μm. Bottom chart exhibits end-point detection of H_2_S production for the corresponding strains in the top chart.

Cysteine degradation, assimilatory sulfite reduction, and dissimilatory sulfate reduction have been reported as sources of H_2_S in bacteria [17,18]. Since the dissimilatory sulfate reduction pathway is absence in *V*. *cholerae* (KEGG pathway: vch00920), H_2_S could only be produced from the first two pathways. As for assimilatory sulfite reduction (KEGG pathway module: vch_M00176), the critical enzyme for H_2_S generation is the NADPH-dependent sulfite reductase, CysJI (Fig 1A), which is assigned to VC0384 (CysJ) and VC0385 (CysI) in *V*. *cholerae* [22]. H_2_S production from cysteine degradation is known to be catalyzed by orthologs of mammalian CBS, CSE and 3MST [17,23]; however, these enzymes have not been identified for *V*. *cholerae*.

To characterize the cysteine degradation pathway for H_2_S biogenesis in *V*. *cholerae*, we first predicted functional proteins by their sequence homology with the CBS, CSE and 3MST in *Homo sapiens* using DELTA-BLAST [24]. As a result, multiple homologous proteins were identified for CBS (VC1061, VC0968, and VC0537) and CSE (VC2683 and VC1671), while only one was found for 3MST (VCA0620). We further examined the presence of key active-site residues [17,25–29] by multiple sequence alignment, and designated VC1061, VC2683 and VCA0620 as putative CBS, CSE and 3MST, respectively (S1 Fig). This hypothesis was supported by the H_2_S production in the single-deletion mutants of CBS, CSE and 3MST candidates (S2 Fig). Among the mutants of CBS candidates, only *Δvc1061* showed a significant reduction in H_2_S production from cysteine compared to wild-type (S2 Fig). Surprisingly, none of the mutants of CSE candidates were defective in H_2_S production (S2 Fig). Since CBS, CSE and 3MST may act together in bacteria [17,30], we further investigated the possible cross-talk among the three enzymes by examining the H_2_S generation in single-, double- and triple-deletion of *cbs*, *cse* and *3mst*. All mutants were indistinguishable from wild-type in H_2_S production except for mutants with *cbs* deletion (Fig 1B). The results indicate that deletion of *cbs* is critical in reducing H_2_S production from cysteine in *V*. *cholerae*.

To examine the contribution of cysteine degradation and assimilatory sulfate reduction to H_2_S production in *V*. *cholerae*, we compared the sulfide generation in *Δcbs* to that of the double-deletion mutant of *cysI* and *cysJ*. We found that blocking the assimilatory sulfite reduction, by deletion of *cysI* and *cysJ*, had little effect on H_2_S production, while interfering with cysteine degradation by *cbs* deletion led to a dramatic reduction in H_2_S production (Fig 1B). The phenotypes suggest that degradation of cysteine is the largest contributor of H_2_S in *V*. *cholerae*. Through real-time detection of H_2_S levels in *V*. *cholerae* cells, we found that H_2_S biogenesis positively correlates with the level of *cbs* expression, which was supported by the data from end-point detection of H_2_S production (Fig 1C). This data, collectively, suggests that CBS is the key enzyme for H_2_S production in *V*. *cholerae*.

### CBS-derived H_2_S increases the resistance to H_2_O_2_ in *V*. *cholerae*

**Fig 2:**
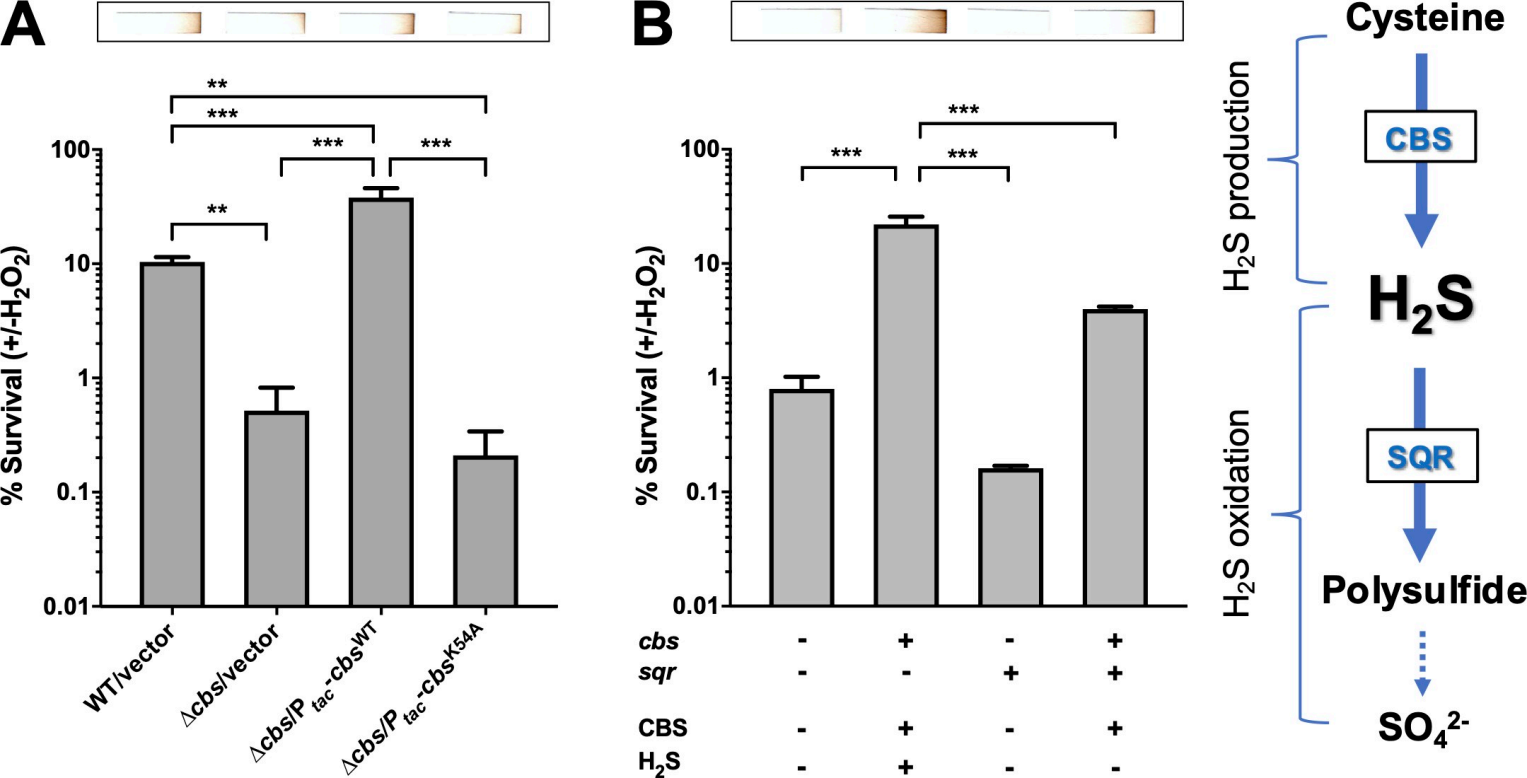
H_2_S is the determinant of H_2_O_2_ resistance caused by *cbs* expression in *V*. *cholerae*. (A). CBS-derived H_2_S renders *V*. *cholerae* cells less susceptible to H_2_O_2_. Bacteria were exposed to 1 mM of H_2_O_2_ for 30 min at their exponential-phase or left untreated in M9 minimal medium (M9 salts plus 2 mM MgSO_4_, 0.1 mM CaCl_2_, and 0.2% casein acid hydrolysate as sole carbon source) containing 200 μM IPTG. Viability was determined by comparing the CFU in the H_2_O_2_-challenged and the unchallenged samples. Top chart exhibited end-point detection of H_2_S production for the corresponding strains in the bottom chart. Three replicates were sampled for each strain. Significance was determined by one-way ANOVA; *p*-value: **, <0.01, ***, <0.001. (B). H_2_S scavenging via the expression of *S*. *aureus sqr* gene impairs the protective effect of *cbs* expression on the survival of *V*. *cholerae*. Left panel: survival under H_2_O_2_ stress were tested for Δ*cbs* strains containing vectors only (*cbs*^*-*^*sqr*^*-*^), either P_*tac*_*-cbs* (*cbs*^*+*^*sqr*^*-*^) or P_*BAD*_*-sqr* (*cbs*^*-*^*sqr*^*+*^), or both of P_*tac*_*-cbs* and P_*BAD*_*-sqr* (*cbs*^*+*^*sqr*^*+*^). Bacteria were grown in M9 minimal medium containing 200 μM of IPTG and 0.02% arabinose and exposed to 1 mM of H_2_O_2_ for 30 min at their mid-log phase. Viability was determined by comparing the CFU in the H_2_O_2_-challenged and the unchallenged samples. Values expressed are means ± S.D. from three experiments. Significance was determined by one-way ANOVA; *p*-value: ***, <0.001. Top chart exhibits end-point detection of H_2_S production for the corresponding strains in the bottom chart in M9 minimal medium. Right panel: design of H_2_S production and elimination system in *V*. *cholerae* cells. In the system, *S*. *aureus sqr* gene was introduced to remove the CBS-derived H_2_S by H_2_S oxidation in order to establish a system with only CBS protein produced.

Carbon source utilization, biofilm formation, virulence induction, and resistance to oxidative stress are important for the survival and colonization of *V*. *cholerae* in aquatic environments and mammalian GI tract. Through analysis of the *cbs* deletion on the above biological functions in *V*. *cholerae*, we found that deletion of *cbs* led to a 10-fold reduction in viability compared to the wild-type under intense H_2_O_2_ stress, while no impact was detected on the other phenotypes (Figs 2A and S3). The impaired viability in the *cbs* mutant was not an artifact resulting from growth defect (Fig D in S3 Fig). In addition, the complement of *cbs* (Δ*cbs/P*_*tac*_*-cbs*) restored cell survival, which further supported the idea that *cbs* expression rendered *V*. *cholerae* cells less susceptible to H_2_O_2_ (Fig 2A).

Since cytoprotective effect of endogenous H_2_S is regarded as a universal defense mechanism against oxidative stress from prokaryotes to mammals [31], we wondered whether CBS-catalyzed production of H_2_S is the principal effector of cytoprotection caused by *cbs* expression. To test this hypothesis, we first disrupted the ability of CBS to produce H_2_S by point mutation and examined the ability of the mutants to survive under H_2_O_2_ stress. With reference to studies on human, *B*. *anthracis*, and *Saccharomyces cerevisiae* [28,29,32,33], we determined that 54 lysine (K) and 241 glutamate (E) residues are the possible key sites for H_2_S production by *V*. *cholerae* CBS. We replaced 54K and 241E with alanine (A) and arginine (R), respectively, and separately overexpressed the mutant CBS proteins in Δ*cbs* cells to examine their enzymatic activity in generating H_2_S. Results showed that in CBS^K54A^-expressing cells, H_2_S production was significantly lower than CBS^WT^-expressing cells, suggesting that K54 residue is critical for H_2_S production (S4 Fig). Then, we compared the viability of the cells expressing CBS^WT^ and CBS^K54A^ under H_2_O_2_ toxicity and found that CBS^K54A^-expressing cells are much more susceptible to H_2_O_2_ than those expressing CBS^WT^ (Fig 2A and Fig A in S5 Fig), which supported the idea that H_2_S is responsible for the cytoprotective effect of CBS expression.

We also validated the importance of CBS-catalyzed H_2_S production in ROS resistance of *V*. *cholerae* using an alternative system. In animals, lethal level of H_2_S is known to be removed mainly via mitochondrial sulfide oxidation, for which the first step is catalyzed by the membrane-bound sulfide:quinone oxidoreductase (SQR) [34]. Bacterial SQRs have been well-studied in photo- and chemoautotrophic bacteria, and recently found to be common in heterotrophic bacteria by an analysis of 4,929 bacterial and 242 archaeal genomes from GenBank [35,36]. *V*. *cholerae* lacks the *sqr* gene, so it cannot oxidize self-produced and exogenous H_2_S [36]. Therefore, we expressed the *sqr* gene of *S*. *aureus* in *V*. *cholerae* via plasmid system to establish strains that produce only CBS protein but no H_2_S (Fig 2B). Complementation of *cbs* in Δ*cbs* restored H_2_S production and reduced cell death by 20-fold under H_2_O_2_ toxicity (Fig B in S5 Fig and Fig 2B, *cbs*^*+*^*sqr*^*-*^ v.s. *cbs*^*-*^*sqr*^*-*^), while CBS-derived H_2_S was partially removed when *cbs* and *sqr* expression were both induced in *Δcbs*, which led to a 10-fold reduction in viability compared with cells that only *cbs* expression was induced (Fig B in S5 Fig and Fig 2B, *cbs*^*+*^*sqr*^*+*^ v.s. *cbs*^*+*^*sqr*^*-*^). *sqr* expression in *Δcbs* further reduced H_2_S generation and impaired the viability of cells by ~80% (Fig B in S5 Fig and Fig 2B, *cbs*^*-*^*sqr*^*+*^ v.s. *cbs*^*-*^*sqr*^-^). This data indicates that H_2_S plays the key role in cell protection, rather than CBS protein.

### CBS-derived H_2_S stimulates the expression of enzymatic ROS scavengers

The chemical nature as a reducing agent endows H_2_S redox buffering capacity [37]. However, the H_2_O_2_ degradation capacity of H_2_S is far below that of catalase or peroxidase [37,38]. In this study, by providing NaHS, the commonly used rapid donor of H_2_S, to the *cbs* deletion mutant, we confirmed the cytoprotection role of H_2_S in *V*. *cholerae*. However, exogenous H_2_S concentration needed to reach millimolar (mM) level to significantly improve cell survival under H_2_O_2_ toxicity, while CBS-derived H_2_S of micromolar (μM) level was adequate to facilitate viability of *V*. *cholerae* (S6 Fig). Therefore, we hypothesized that CBS-derived H_2_S has functions other than direct neutralization of H_2_O_2_, making it more efficient in protecting *V*. *cholerae* cells from H_2_O_2_ stress, and that stimulation of H_2_O_2_ detoxifying enzymes is likely to be one of the mechanisms.

**Fig 3:**
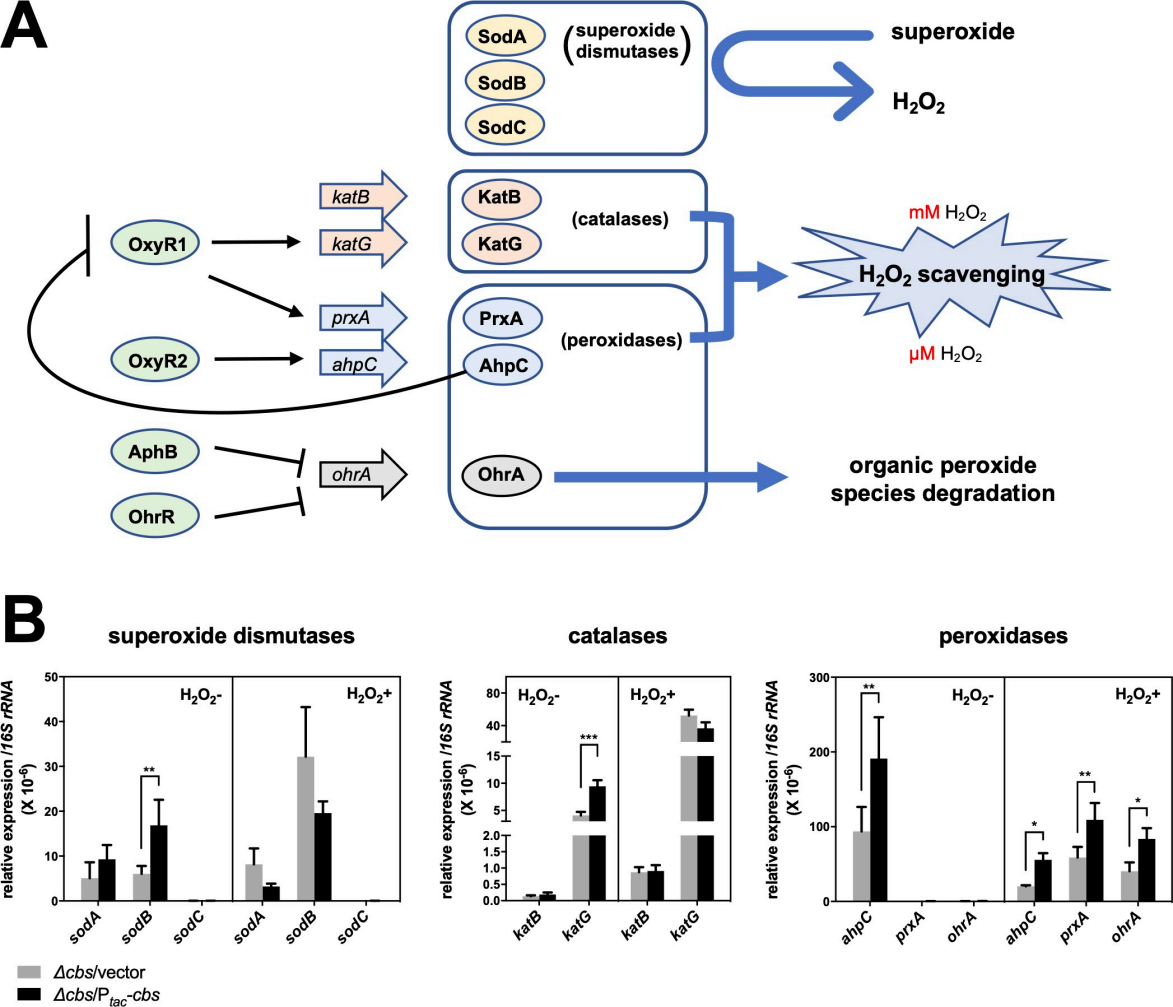
CBS-derived H_2_S stimulates the expression of enzymatic ROS scavengers. (A). Illustration for enzymatic ROS scavengers in *V*. *cholerae*. Up to date, there are three SODs, VC2694 (SodA), VC2045 (SodB), and VC1583 (SodC), two catalases, VC1585 (KatB) and VC1560 (KatG), and three peroxidases, VC0731 (AhpC), VC2637 (PrxA), and VCA1006 (OhrA), reported for *V*. *cholerae* [6–9]. Expression of *katG* and *prxA* are positively regulated by transcription factor VC2636 (OxyR1), while *ahpC* expression is activated by VC0732 (OxyR2), and in turn inhibits OxyR1 expression [9]. SODs detoxify superoxide and generate H_2_O_2_. OhrA specifically degrades organic peroxide species. Peroxidases are the primary scavengers confronting low micromolar (μM) level of H_2_O_2_, while catalase activity predominates at millimolar (mM) levels of H_2_O_2_ [4]. (B). *cbs* expression and enzymatic antioxidants expression. The relative expression of SODs, catalases, and peroxidases in exponentially grown *cbs* deletion mutant (Δ*cbs*/vector), and *cbs* overexpression strain (Δ*cbs/*P_*tac*_*-cbs*), with or without H_2_O_2_ treatment (1 mM, 10 min), was examined by real-time qPCR. *cbs* expression was induced by 200 μM of IPTG. Values expressed are means ± S.D. from three experiments. Significance was determined by *t*-test; *p*-value: *, <0.05, **, <0.01, ***, <0.001.

In *V*. *cholerae*, the reported ROS degrading enzymes include three SODs, SodA, SodB, and SodC, two catalases, KatB and KatG, and three peroxidases, glutathione peroxidase PrxA, alkyl hydroperoxide reductase AhpC, and organic hydroperoxide resistance protein OhrA [6–9]. OhrA specifically degrades lipid peroxides into unreactive alcohols, and SODs destroy free radical superoxide by converting it to H_2_O_2_, while the other enzymes can use H_2_O_2_ as substrate (Fig 3A). To investigate whether H_2_S regulates the enzymatic antioxidant system, we first examined the expression of these ROS detoxifying enzymes in *V*. *cholerae* cells with *cbs* overexpression or deletion using RT-qPCR. Results showed that *sodB*, *katG*, and *ahpC* were expressed significantly higher in the *cbs* overexpression strain compared to *cbs* mutant (Fig 3B, H_2_O_2_^-^). The up-regulation of KatG was confirmed at the translational level by proteomic analyses on *cbs-*deficient and overexpressed cells (S3 Table). In addition, the proteomic data revealed that *V*. *cholerae* cells with higher levels of endogenous H_2_S produced more of the other two enzymatic antioxidants, i.e., the DNA-binding protein from starve cells (DPS) (VC0139) which protects DNA from ROS damage by direct binding to DNA [39], and the homolog of thioredoxin 1 (Trx1) (VC0306) which may function as protein redox regulator [40]. HupA (VC0273), RhlB (VC0305) and VC0105, which function in DNA stabilization, RNA degradation, and protoporphyrin-IX biosynthesis, respectively, might be targets of Trx1, as illustrated by STRING network (S7 Fig). The enhanced production of these enzymatic antioxidants should endow *V*. *cholerae* cells with a higher tolerance to oxidative stress. These results support the idea that CBS-catalyzed production of H_2_S can protect *V*. *cholerae* cells via stimulating H_2_O_2_ detoxifying enzymes.

### CBS-derived H_2_S enhances KatB activity via protecting iron levels in protein

To fully understand the cytoprotective mechanism of CBS-derived H_2_S, we further investigated the impact of *cbs* expression on the expression pattern of enzymatic antioxidants under H_2_O_2_ toxicity. Confronting H_2_O_2_, expression of *sodB*, *katB*, and *katG* was up-regulated, but with no regard to *cbs* expression level, while *ahpC* was down-regulated but remained higher level in *cbs* overexpression strain (Fig 3B, H_2_O_2_^+^). As to *prxA* and *ohrA*, up-regulation of expression was detected with relatively higher expression in *cbs*-overexpressing cells (Fig 3B, H_2_O_2_^+^).

**Fig 4:**
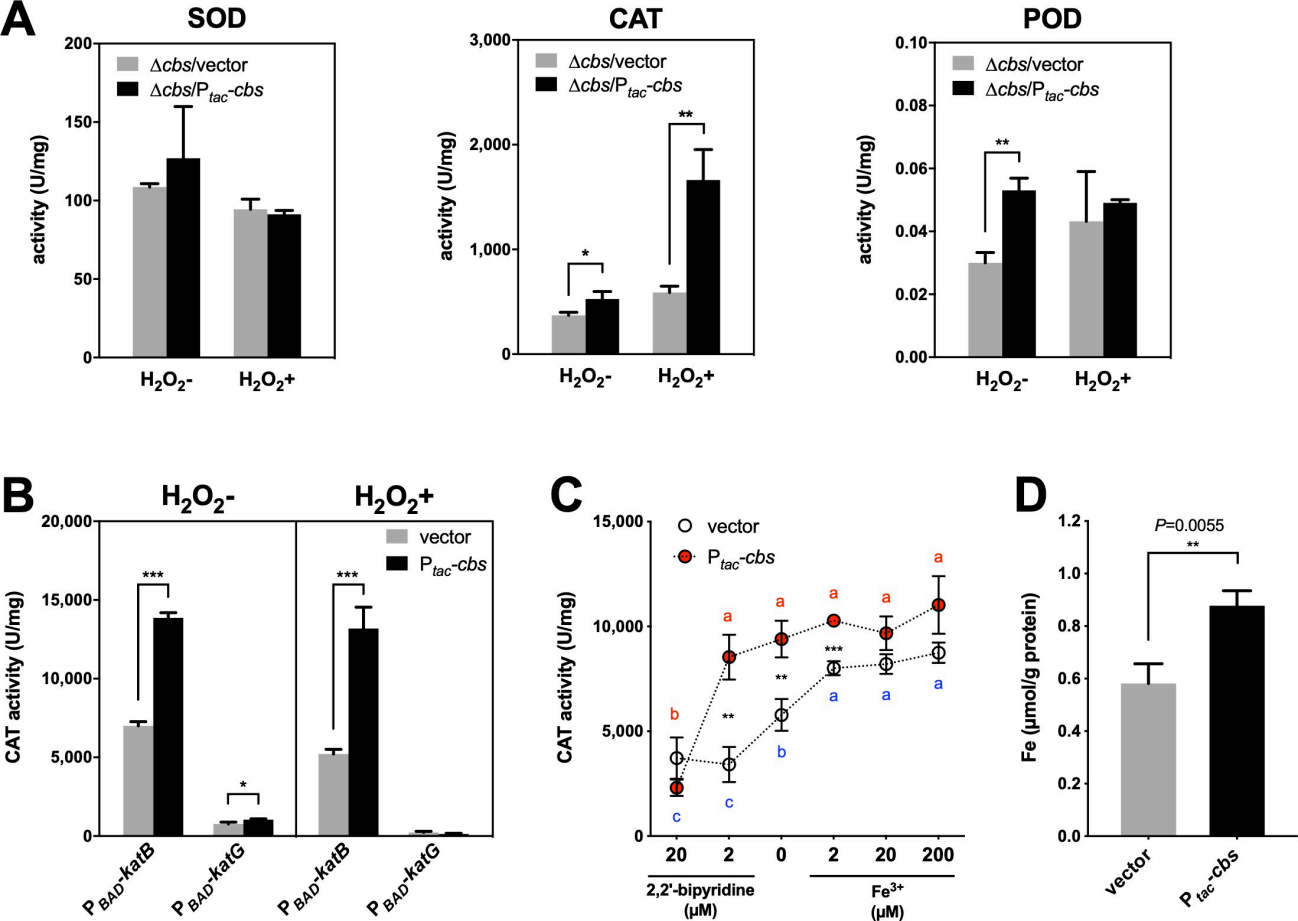
CBS-derived H_2_S enhances catalase activity in *V*. *cholerae*, which relates to the facilitation of cellular iron content. (A). Contribution of superoxide dismutases, catalases and peroxidases expression to enzymatic activity in H_2_O_2_ detoxification. Superoxide dismutase (SOD), catalase (CAT) and peroxidase (POD) activity in crude extracts of *cbs*-deficient (Δ*cbs*/vector) and overexpressed (Δ*cbs*/P_*tac*_*-cbs*) cells. Cells were grown in M9 minimal medium (M9 salts plus 2 mM MgSO_4_, 0.1 mM CaCl_2_, and 0.2% casein acid hydrolysate as sole carbon source), containing 200 μM of IPTG, and challenged with or without H_2_O_2_ (1 mM, 20 min) at their mid-log phase. Values expressed are means ± S.D. from three experiments. Significance was determined separately for the H_2_O_2_^-^ and H_2_O_2_^+^ samples by *t*-test; *p*-value: *, <0.05, **, <0.01. (B). Impact of *cbs* expression on the catalase activity of KatB and KatG, respectively. *katB* and *katG* was separately expressed under *P*_*BAD*_ inducible promoter in the triple-deletion mutant of *cbs*, *katB* and *katG*. Catalase activity in crude extracts of samples with induced *cbs* expression (P_*tac*_*-cbs*) was compared with that of *cbs*-deficient cells (pMal-c2x vector). Bacterial cultures were treated with or without H_2_O_2_ (1 mM, 20 min) before collection. Values are means ± S.D. from three experiments. Significance was determined by *t*-test; *p*-value: ns, not significant, *, <0.05, ***, <0.001. (C). CBS-derived H_2_S helps to retain the catalase activity of KatB under iron-deficient conditions. Cells were grown in M9 medium containing 0.2% casein acid hydrolysate as the sole carbon source and induced with 0.5 mM IPTG and 0.02% arabinose. Iron chelator 2,2’-bipyridine or FeCl_3_ was added to monitor the iron content of the medium as indicated. Catalase activity in crude extracts of *ΔcbsΔkatBΔkatG* cells, containing P_*BAD*_*-katB*, and having either P_*tac*_*-cbs* (*cbs*^+^) or vector control (*cbs*^-^), was examined. Significance was determined by two-way ANOVA from the data of three independent experiments. Significant differences in the mean rank of the catalase activity of each strain at different iron levels are shown in alphabetical order (*cbs*^*-*^, blue letters; *cbs*^*+*^, red letters), with the same letter indicating a *p*-value > 0.05. The significant differences between *cbs*^*-*^ and *cbs*^*+*^ strains at certain 2,2’-bipyridine concentrations were also indicated; *p*-value, **, <0.01, ***, <0.001. (D). Impact of *cbs* expression on the iron content in KatB proteins. Hig-tagged *V*. *cholerae* KatB was expressed under pBAD promoter in triple-deletion mutant of *cbs*, *katB* and *katG*, with (P_*tac*_-*cbs*) or without (vector) additional *cbs* expression. Strains were cultured and induced in M9 minimal medium (M9 salts plus 2 mM MgSO_4_, 0.1 mM CaCl_2_, and 0.2% casein acid hydrolysate as sole carbon source). Purified but not desalted KatB protein samples were subjected to quantification of iron content based on chromogenic reaction with ferrozine (cat. number R22185, Shanghai yuanye Bio-Technology). Values expressed are means ± S.D. from three experiments. Significance was determined by *t*-test; *p*-value: **, <0.01.

We further assessed the relative contribution of transcriptional up-regulation of SODs, CATs, and PODs to H_2_O_2_ detoxification by examining the SOD, CAT and POD activity respectively in crude enzyme extracts of *V*. *cholerae* cells. Surprisingly, although *prxA* and *ohrA* expression were significantly induced by H_2_O_2_ and higher in *cbs*-overexpressed cells, they made no contribution to the H_2_O_2_ degradation capacity of cells (Fig 4A, POD). Similarly, up-regulation of *sodB* did not lead to increased SOD activity (Fig 4A, SOD). On the contrary, catalase expression conferred a strong ability in destroying H_2_O_2_ to the cells (Fig 4A, CAT). Particularly, in the presence of H_2_O_2_, the *cbs* overexpression strain showed higher CAT activity, while it displayed no difference in catalase expression compared with *cbs* mutant (Figs 3B and 4A). This implied that *cbs* expression might enhance the activity of catalases at the post-translational level. To evaluate the contribution of CBS-derived H_2_S to the enhanced CAT activity under *cbs* expression, we again co-expressed the *sqr* gene of *S*. *aureus* and the *cbs* gene of *V*. *cholerae* in *Δcbs*, and examined their impact on CAT activity. As a result, the enhancement of CAT activity by *cbs* expression was abolished by *sqr* expression (S8 Fig). This result supports the idea that H_2_S is the determinant of the enhanced CAT activity caused by *cbs* expression, consistent with its roles in the promoted viability.

The two catalases in *V*. *cholerae*, KatB and KatG, are presumed to have differences in enzymatic characteristics; according to KEGG Orthology, KatB is defined as a monofunctional catalase (K03781; EC 1.11.1.6), which has CAT activity only, while KatG is defined as a bifunctional catalase (K03782; EC 1.11.1.21), which can exhibit both CAT and POD activity [41]. By overexpressing *katB* and *katG* in triple-mutant of *cbs*, *katB*, and *katG*, respectively, via a plasmid system, we examined the impact of *cbs* expression on the CAT activity in *V*. *cholerae* cells with fixed catalase expression. In the absence of H_2_O_2_, cells expressing only KatB (P_*BAD*_*-katB*) or KatG (P_*BAD*_*-katG*) both exhibited higher CAT activity when *cbs* was co-expressed (Fig 4B). When H_2_O_2_ was imposed, the differences in CAT activity between *cbs-*deficient and overexpressed cells was observed only in KatB (Fig 4B). Therefore, we presumed that KatB was the preferred target of CBS-derived H_2_S.

Being a heme-catalase, KatB (EC 1.11.1.6) depends on its co-factor, heme, for the enzymatic activity of reducing H_2_O_2_ to H_2_O [41]. However, the production of monomeric apo-catalase does not rely on heme [42]. Given that iron availability has no impact on catalase expression in *V*. *cholerae* [43], we hypothesized that iron content would affect catalase activity at the post-translational level, which could be partially compensated by CBS-derived H_2_S. To test this hypothesis, we examined the impact of *cbs* expression on KatB activity in *V*. *cholerae* cells under iron-deficient (achieved by supplementation of iron chelator 2,2’-bipyridine) or iron-enriched (achieved by supplementation of FeCl_3_) conditions. As expected, the KatB activity in *cbs*-deficient cells decreased rapidly with increasing iron chelator concentration, whereas *cbs* expression retarded the deceleration in the activity of KatB under iron deficiency (2 μM 2,2’-bipyridine), allowing the *cbs*-overexpression strain to exhibit higher catalase activity compared to the *cbs* mutant, while iron-rich (20 and 200 μM Fe^3+^) conditions overwhelmed the protective effect of *cbs* expression on KatB activity (Fig 4C). However, *cbs* expression failed to rescue catalase activity under iron starvation condition (20 μM 2,2’-bipyridine) (Fig 4C). To further investigate the hypothesis that *cbs* expression protects KatB activity by maintaining iron levels, we compared the iron content in KatB proteins expressed in cells with different *cbs*-expression levels, and consequently detected significant higher iron content in KatB protein expressed in *cbs*-overexpressed cells (Figs 4D and S9).

### CBS-dependent ROS resistance works through promoting iron storage

**Fig 5:**
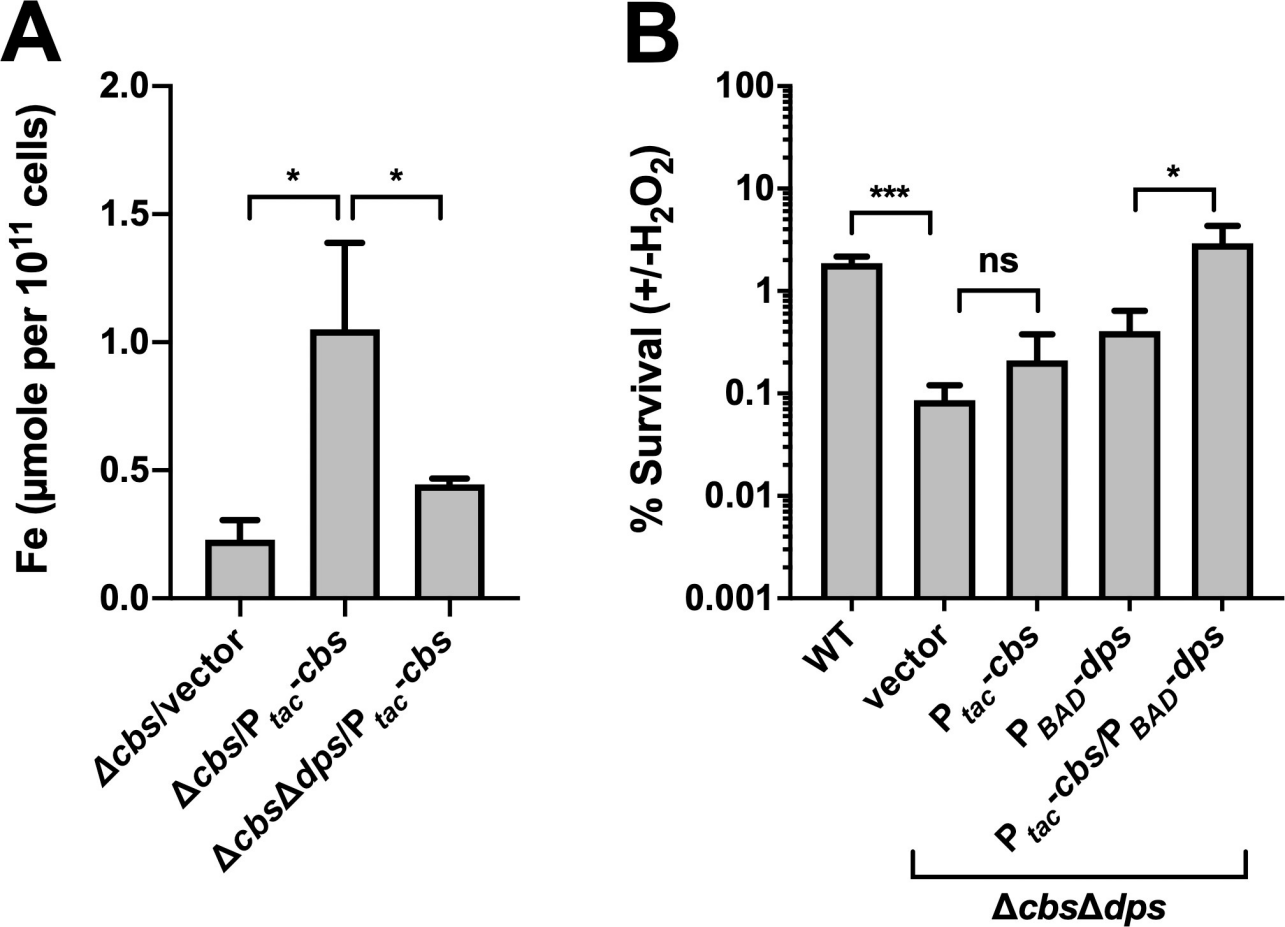
CBS-dependent ROS resistance works through promoting iron storage. (A). *cbs* expression promotes cellular iron storage. Cells were obtained in M9 minimal medium with 0.2% casein containing 0.5 mM IPTG and 0.02% arabinose. Iron content was assessed with ICP-MS and normalized with cell numbers. Values expressed are means ± S.D. from three experiments. Significance was determined by *t*-test; *p*-value: *, <0.05. (B). *cbs* expression protects cells from H_2_O_2_ in iron-deficient conditions. Bacteria were exposed to 1 mM of H_2_O_2_ for 30 min at their exponential-phase or left untreated in M9 minimal medium (M9 salts plus 2 mM MgSO_4_, 0.1 mM CaCl_2_, and 0.2% casein acid hydrolysate as sole carbon source). Viability was determined by comparing the CFU in the H_2_O_2_-challenged and the unchallenged samples. Three replicates were sampled for each strain. Significance was determined by one-way ANOVA; *p*-value: *, <0.05; ***, <0.001; ns, not significant.

The pattern of *cbs* expression regulating KatB activity suggested that *cbs*-dependent ROS resistance may be achieved by promoting cellular iron storage. To investigate this hypothesis, we first examined the effect of *cbs* expression on whole-cell iron content of *V*. *cholerae* and explored whether this was achieved by affecting the cellular iron storage machinery. DPS is likely to be a target of *cbs* expression in regulating intracellular iron content, since DPS proteins are known to function in iron storage-detoxification [39,44,45], and our proteomic data indicated that DPS (VC0139) is highly expressed in *cbs*-overexpressing cells. Therefore, we examined the iron content in *cbs*-overexpressing and deficient cells in the presence or absence of *dps* expression using ICP-MS, and consequently found that *cbs* expression conferred higher iron content to the cells, but *dps* deletion in turn impaired cellular iron content (Fig 5A). These results supported the idea that CBS-derived H_2_S promotes iron fixation and confirmed the role of DPS in iron storage.

We further investigated whether *cbs* expression promotes oxidative resistance through iron sequestration by comparing the viability of *cbs* overexpression and deletion cells under *dps*-deficient or overexpression conditions. Results showed that *dps* deletion eliminated *cbs*-dependent effect on H_2_O_2_ resistance, which was not influenced by *dps* overexpression (Fig 5B). Therefore, the idea that CBS-derived H_2_S promotes oxidative resistance through iron storage was supported.

### *cbs* expression alters the fitness of *V*. *cholerae* in host via modified susceptibility to oxidative stress

**Fig 6:**
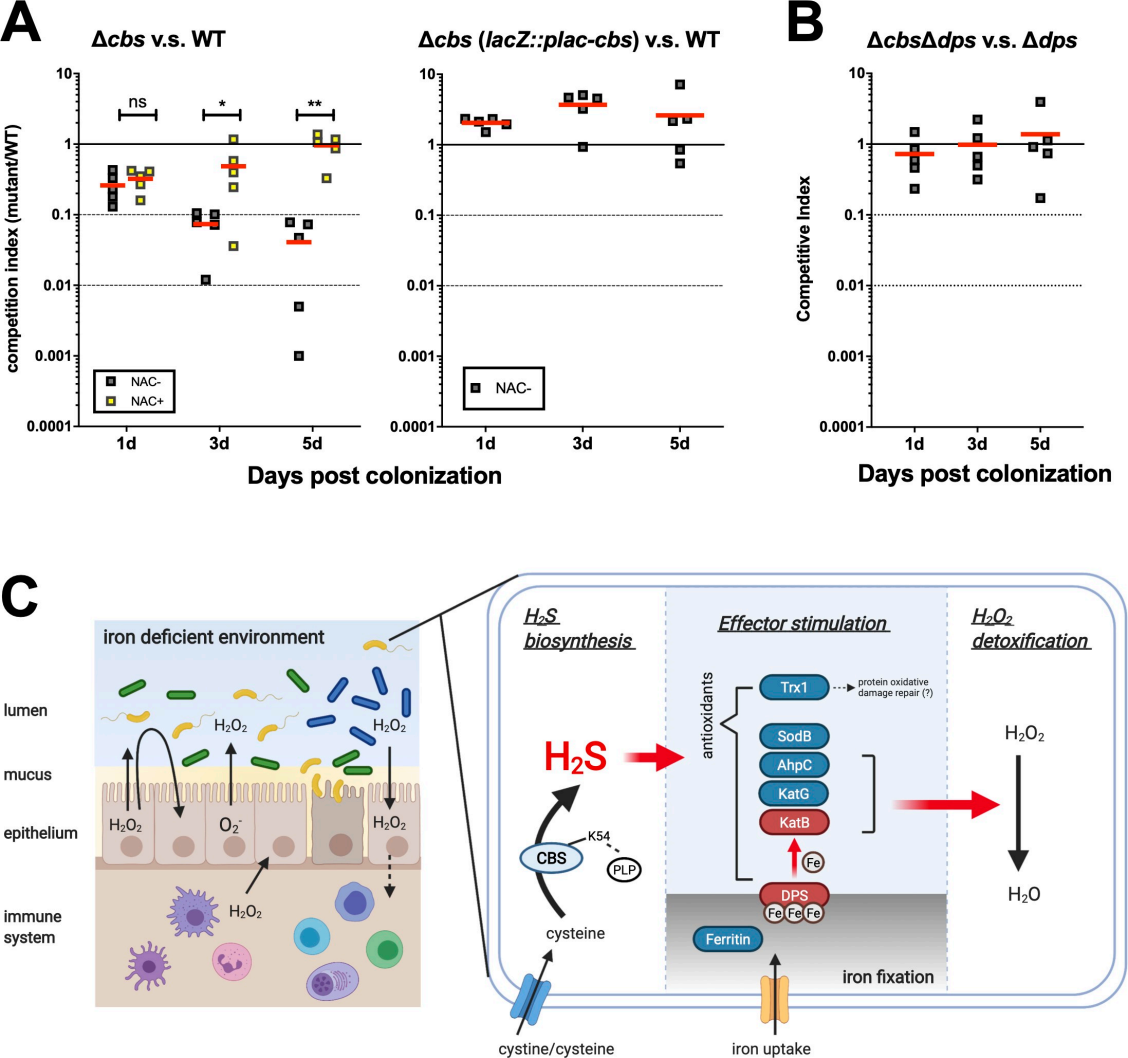
CBS-derived H_2_S promotes the fitness of *V*. *cholerae* in adult mouse via fighting against oxidative stress. (A). Adult CD1 mice were treated, without or with 1% N-acetyl cysteine (NAC), in drinking water for 7 days, then administrated with wild-type (WT) and either Δ*cbs* or Δ*cbs* complemented by single copy *P*_*lac*_*-cbs* (Δ*cbs lacZ*::*P*_*lac*_*-cbs*) mutants after streptomycin treatment. Fecal pellets were collected at 1, 3, and 5 days post inoculation. Bacterial loads were quantified by plating. Competition index (CI) was calculated as the ratio of mutant to wild-type colonies normalized with the input ratio. Red line indicated for the average CI. Significance was determined by *t*-test; *p*-value: ns, not significant, *, <0.05, **, <0.01. (B). Streptomycin-treated adult CD1 mice were administrated with Δ*dps* and Δ*cbs*Δ*dps*. Fecal pellets were collected at 1, 3, and 5 days post inoculation. Bacterial loads were quantified by plating. Competition index (CI) was calculated as the ratio of Δ*cbs*Δ*dps* to Δ*dps* colonies normalized with the input ratio. Red line indicated for the average CI. (C). A proposed mechanism for the cytoprotective effects of CBS-derived H_2_S against oxidative stress in *V*. *cholerae*. Iron availability is limited for *V*. *cholerae* during infection of host. The *cbs*-dependent generation of H_2_S from cysteine stimulates the expression of several antioxidants and facilitates the acquisition and storage of iron in *V*. *cholerae*. Enriched intracellular iron pool promotes the activity of heme-catalase KatB, by contributing to the assembly of active catalases. The illustration is created with BioRender.com.

To verify the impact of CBS-derived H_2_S on colonization and fitness of *V*. *cholerae* in mammalian host, we used ROS-level-controlled adult mice models to address this issue: 1) high ROS level, streptomycin treatment only; 2) low ROS level, streptomycin plus N-acetylcysteine (NAC) treatment [7]. All the chemicals were supplied via drinking water. In the high ROS model, the *cbs* mutant (Δ*cbs*) was outcompeted by wild-type (WT), while complementation of *cbs* (Δ*cbs lacZ*::*P*_*lac*_*-cbs*) exhibited similar survival as wild-type; in the low ROS model, the impaired fitness of *Δcbs* was no longer observed (Fig 6A). We also evaluated the association of *cbs*-dependent ROS resistance with iron storage *in vivo*, based on competition assay of Δ*cbs*Δ*dps* and Δ*cbs* in streptomycin-treated adult mice. The result demonstrated that the *cbs* mutant displayed no colonization defect when the iron storage machinery DPS was removed (Fig 6B). These results together suggested a positive correlation between *V*. *cholerae* fitness and *cbs* expression under oxidative stress in the host environment and provided *in vivo* evidence for the idea that *cbs* expression ensures ROS resistance by promoting iron storage. We also assessed infant mouse colonization for *cbs* mutant (S10 Fig). However, compared with wild-type, *cbs* mutant showed no defects in colonizing infant mice, which was thought to be due to the lack of inflammation in infant mouse intestine, resulted from relative immaturity of the immune system [46] and the insufficient exposure to selective pressures during the short-term colonization of the infant mouse intestine (18 hrs).

To sum up, in *V*. *cholerae*, the main source of H_2_S is CBS-catalyzed degradation of cysteine. H_2_S can alleviate oxidative stress via 1) stimulating the expression of antioxidants, including ROS scavengers KatG, AhpC and SodB, the DNA protective protein DPS, and the protein redox regulator Trx1, and 2) promoting H_2_O_2_ detoxification capacity, particularly by maintaining KatB activity at the post-translational level. The up-regulation of catalase activity is achieved through enhanced iron storage. Such cytoprotective roles against ROS works in the iron-deficient host intestine as well, resulting in improved colonization of *V*. *cholerae* in an adult mouse model (Fig 6C).

## Discussion

### Endogenous H_2_S production in *V*. *cholerae*

Cysteine catabolism is the main source of endogenous H_2_S, which involves mainly three enzymes in mammals, CBS and CSE of the transsulfuration pathway that produce H_2_S predominantly from cysteine, and 3MST that uses the 3-mercaptopyruvate produced from cysteine via cysteine aminotransferase as a substrate. Orthologs of the three enzymes are found in the majority of bacterial species by analyzing genomic data; however, CBS/CSE and 3MST generally do not function together in the same bacterial species [17]. For example, *B*. *anthracis*, *P*. *aeruginosa*, and *S*. *aureus* utilize CBS and CSE in H_2_S production, whereas CSE and 3MST are both required for *Shewanella oneidensis*, and 3MST alone works for *E*. *coli* [17,30]. Specifically, anaerobic cysteine-catabolism via CyuA has been reported in *Salmonella enterica* and *E*. *coli* [47]; however, *V*. *cholerae* does not have a CyuA homolog. In this study, we found that endogenous H_2_S production from cysteine mainly relies on CBS in *V*. *cholerae*, not CSE or 3MST (Fig 1B); thus, proposing a preference for CBS in cysteine degradation which has not been reported for other bacteria. The ability of *V*. *cholerae* CBS to produce H_2_S by degrading self-derived cysteine could be detected in the absence of exogenous cysteine, and the H_2_S produced could also function in cell protection (S11 Fig).

Apart from cysteine metabolism, dissimilatory sulfate reduction and assimilatory sulfite reduction also contribute to H_2_S production in bacteria [18]. However, *V*. *cholerae* lacks the complete pathway of dissimilatory sulfate reduction (KEGG pathway: vch00920); meanwhile, assimilatory sulfite reduction contributes to a very small fraction of H_2_S produced in *V*. *cholerae*, as revealed in this study (Fig 1B). Therefore, we speculate that the H_2_S release detected in *cbs* deletion mutant is probably attributed to a combination of assimilatory sulfite reduction, and endogenous non-enzymatic production of H_2_S from glucose, sulfur-containing amino acids, polysulfides, and elemental sulfur, or even non-enzymatic degradation of cysteine in experiments [48,49].

### CBS-derived H_2_S enhances catalase activity via promoting iron storage

As we demonstrated in this study, stimulation of catalase expression and activity is an important antioxidant mechanism in *V*. *cholerae*. The stimulation of catalase activity by endogenous H_2_S is observed in *E*. *coli*, but the mechanism is not clear [17].

H_2_S has been reported to modify protein function by persulfidation of protein cysteine residues, which can serve as a potential protecting mechanism of thiol residues towards oxidative stress [50]. Based on persulfide proteomic analysis, catalase has been identified as a target of H_2_S in *Arabidopsis thaliana* and mammalian cells [14,51]. However, *in vitro* studies using NaHS as a H_2_S donor suggested an inhibitory effect of H_2_S on catalase activity [52]. Moreover, since *V*. *cholerae* KatB possesses no cysteine residues (GenBank accession: WP_000551118), it is not possible that H_2_S modifies KatB directly via persulfidation.

Another way that H_2_S directly regulates protein activity is through interactions with protein metal centers, especially for heme-proteins, which includes coordination, reduction of iron centers, and formation of sulfheme derivatives [50]. However, sulfcatalase is the inhibitory derivative of heme-catalase, which is generated through the reaction of catalase, H_2_S and H_2_O_2_ [53]. Up to date, phosphorylation is the only PTM known to enhance CAT activity in mammals and plants [54]. Further investigation is needed on whether CBS-derived H_2_S indirectly regulates catalase activity by promoting phosphorylation.

As heme-proteins, *V*. *cholerae* KatB and KatG are presumed to rely on their co-factor heme for enzymatic activity. As shown in Fig 4C, we had demonstrated that *cbs* expression could help *V*. *cholerae* cells to retain the CAT activity of KatB under iron-limiting conditions. Such a protective effect on CAT activity was also observed in KatG (S12 Fig). However, the CAT activity of KatG was obviously impaired under H_2_O_2_ stress which may result from its relatively low tolerance to H_2_O_2_ [41,55] and showed no difference between *cbs* mutant and *cbs*-overexpressing strain (Fig 4B). Since CBS-derived H_2_S enhanced KatB activity under both H_2_O_2_ toxicity and iron-deficiency (Fig 4B and 4C), we further investigated the relationship between KatB activity and iron content in purified KatB protein and found that *cbs* expression positively regulated the iron level in KatB protein (Fig 4D). Using ICP-MS analysis, we confirmed that cells with higher H_2_S levels processed greater iron content (Fig 5A and Fig A in S5 Fig), which was no longer observed in the absence of iron storage machinery such as DPS protein (Fig 5A), and therefore proposed that CBS-derived H_2_S can indirectly enhance the activity of heme-containing catalases by facilitating cellular iron storage.

Since access to iron in the host intestine is limited due to iron sequestration by host iron-binding proteins and competition with commensal bacteria [56], the ability of H_2_S to promote iron uptake can work as a supplement to the enhancement of catalase activity, and further improve the fitness of *V*. *cholerae* in the host environment.

### Greater cytoprotective effect of CBS-derived H_2_S compared to exogenous H_2_S

Our data suggest different modes of endogenous and exogenous H_2_S in resisting H_2_O_2_ in *V*. *cholerae*, as exogenous H_2_S needs to reach mM levels to exert the cytoprotective effect of CBS-derived H_2_S at μM levels (S6 Fig). Studies on *E*. *coli* also showed that endogenous H_2_S produced from cysteine degradation can stimulate catalase activity and protect cells from oxidative stress [17], while exogenous H_2_S mimicked by NaHS inhibits the activity of catalase and further inhibits growth of *E*. *coli* through oxidative damage [57]. Furthermore, since over 80% of the H_2_S in a biological system exists in the form of hydrosulfide ion (HS-), which is not completely free to diffuse, channels for HS- transport may exist in *V*. *cholerae*, as already identified in *Clostridium difficile* [58].

## Materials and methods

### Ethics statement

All animal experiments were carried out in strict accordance with the animal protocols that were approved by the Ethical Committee of Huazhong University of Science and Technology (Permit Number: SYXK (E) 2016–0057).

### Strains, plasmids and growth conditions

A list of all bacterial strains and plasmids used in this study is given in S1 Table. All *V*. *cholerae* strains used in this study were derived from El Tor C6706 [59] and propagated in LB media containing appropriate antibiotics (100 μg/ml for streptomycin, 50 μg/ml for kanamycin, 100 μg/ml for ampicillin) at 37°C unless otherwise noted. In-frame deletion of *V*. *cholerae* mutants were constructed by cloning the regions flanking the target gene into the suicide vector, pWM91, containing a *sacB* counter-selectable maker [60]. The resulting plasmids were introduced into *V*. *cholerae* by conjugation, and deletion mutants were selected for double homologous recombination events. Chromosomal complementation of *cbs* (VC1061) was constructed by inserting *P*_*lac*_*-cbs* into the *lacZ* locus using the suicide vector, pJL1 [61]. The plasmid overexpression *cbs* was constructed by cloning *cbs* coding region downstream of the *tac* promoter of pMal-c2x (New England Biolabs). Plasmids containing *P*_*BAD*_ inducible promoter for overexpressing *katB* (VC1585), *katG* (VC1560), *dps* (VC0139), or *sqr* were constructed by cloning the coding region of target genes into pACYC177 [62]. The *sqr* gene fragment was amplified from the genome of *Staphylococcus aureus* (MRSA, ATCC 43300).

### Detection of H_2_S

End-point detection of H_2_S production during growth was conducted with lead acetate paper strips (cat. number W-012601, Shanghai SSS Reagent) in test tubes. Overnight cultures of test strains were 1:100 sub-cultured into fresh medium, followed by incubation at 37°C, 200 rpm for 6 hrs. For experiments performed in Luria-Bertani broth without cysteine supplement and in M9 medium with glucose as the sole carbon source, cultures of test strains were further incubated overnight at 37°C without shaking. Before incubation, lead paper strips were fixed to the inner wall of test tubes, above the liquid surface of test strains. Stained paper strips were scanned for quantification of H_2_S production as previously described [17,19]. NaHS standard was applied for semi-quantification of H_2_S as previous described [63]. The calibration curve for sulfide was Y = 3×10^6^ x—3955.4 (R^2^ = 0.9993), for X representing the amount of sulfide (μmole) and Y representing the integrated density of gray values measured with ImageJ software.

Real-time detection of intracellular H_2_S level was performed with the WSP-5 fluorescent probe (cat. Number 1593024–78–2, Cayman chemicals), with the following protocol modified from Peng et al. (2014) [64]. Briefly, the WSP-5 probe was added into log-phase bacterial culture for a final concentration of 15 μM, followed by stationary incubation at 37°C for 1 hr. The extra probe was removed by centrifugation, and bacterial cells were resuspended with PBS buffer for microscopic observation. Fluorescent signals were examined at 488–524 nm and images were taken with an inverted fluorescence automated live cell microscope (Olympus IX81) equipped with a 100 × oil objective and an EMCCD (iXon 897i Andor) was applied to continuously record the bright-field and confocal images.

### Generation of growth curves

Overnight cultures of test strains were 1:1,000 sub-cultured in LB and grown to log-phase at 37°C with shaking. Bacterial cultures were pelleted by centrifugation, washed once and adjusted OD_600_ to 0.1 with fresh medium. Growth kinetics of the test strains were determined by OD_600_ measurement in 96-well microplate using microplate-reader (Spark, Tecan). Samples were incubated at 37°C with shaking, and readings were taken every 20 min for 16 hrs. Four individual experiments were taken, and the mean value was plotted.

### Susceptibility to H_2_O_2_ *in vitro*

Overnight cultures of test strains were 1:1,000 sub-cultured into M9 minimal medium (M9 salts plus 2 mM MgSO_4_ and 0.1 mM CaCl_2_), containing 0.2% casein acid hydrolysate, and grown at 37°C, 200 rpm until mid-log phase. In the case that inductive agents (i.e. Isopropyl-β-D-thiogalactoside (IPTG) or arabinose) were needed, they were supplied when OD_600_ reached approximately 0.1. For tests on exogenous H_2_S, the rapid H_2_S donor, NaHS, was supplied to exponential-phase cultures 1 hr before H_2_O_2_ treatment. Briefly, H_2_O_2_ challenge was conducted by adding H_2_O_2_ directly into exponential-phase cultures for appropriate concentrations, followed by stationary incubation at 37°C for 30 min. Serial dilutions of the challenged samples were plated on LB agar for an enumeration of viable cells. Survival rate was calculated for each strain by comparing the CFU in the H_2_O_2_-challenged and the unchallenged samples.

### Gene expression analysis via qPCR

Gene expression was examined for *V*. *cholerae* strains with or without H_2_O_2_ challenge.

#### RNA extraction

*V*. *cholerae* strains were grown in M9 minimal medium, containing 0.2% casein acid hydrolysate as sole carbon source, until mid-log phase. In the case that inductive agents (i.e. IPTG or arabinose) were needed, they were supplied one hour after the start of incubation. Cultures were then treated with or without 1 mM H_2_O_2_ for 10 min at 37°C. Total RNA was extracted with the Bacteria RNA Extraction Kit (cat. number R403-01, Vazyme), according to the manufacturer’s protocol.

#### cDNA synthesis

About 500 ng total RNA was applied to gDNA digestion and subsequent first-strand cDNA synthesis with gene-specific primers (S2 Table), using HiScript II 1^st^ Strand cDNA Synthesis Kit (cat. number R212, Vazyme).

#### qPCR

Each real-time PCR reaction system (20 μl) contained 10 μl of 2×T5 Fast qPCR Mix (SYBR Green I) (cat. number TSE202, TSINGKE), 7.4 μl of ddH_2_O, 0.8 μl of 10 μM forward primer, 0.8 μl of 10 μM reverse primer, and 1 μl of 10-fold dilution of the reverse transcription system. All the primers had been examined for PCR specificity and efficiency. The *16S rRNA* gene was used for normalization. Amplification was carried out using CFX Connect Real-time Detection System (Bio-Rad) with the following program: initial denaturation at 95°C for 1 min, followed by 40 cycles of amplification, including denaturation at 95°C for 10 sec, annealing at 55°C for 5 sec, and extension at 72°C for 15 sec (+ plate read), which ended with the generation of a melting curve. Experiments were performed on three biological replicates. Results were presented with the average data, error bars corresponded to the standard deviation.

### Determination of superoxide dismutase, catalase and peroxidase activity

Overnight cultures of *V*. *cholerae* strains were 1:1,000 sub-cultured into fresh medium and grown at 37°C 200 rpm until log phase. Inductive agents (i.e. IPTG or arabinose) were supplied one hour after the start of incubation when needed. H_2_O_2_ treatment (1 mM, 20 min) was performed on the mid-log cultures when indicated. When treatment ended, bacterial cells were rinsed with pre-cooled PBS buffer, resuspended with pre-cooled sonication buffer (50 mM potassium phosphate buffer (pH 7.0) containing 10% (v/v) glycerol, 25 μM PMSF), and then lysed with sonication. The lysates were subjected to analysis of superoxide dismutase and catalase activity using the Total Superoxide Dismutase Assay Kit (cat. number S0101, Beyotime Biotechnology) and the Catalase Assay Kit (cat. number S0051, Beyotime Biotechnology), respectively, according to the manufacturer’s protocol. One unit of superoxide dismutase was defined as the amount of enzyme needed to exhibit 50% dismutation of the superoxide radical generated by xanthine oxidase and hypoxanthine. And one unit of catalase was defined as the amount of enzyme required for the decomposition of 1.0 μmole of hydrogen peroxide within one minute at pH 7.0 at 25°C. For analysis of peroxidase activity, each reaction system contained 210 μl of ddH_2_O, 32 μl of potassium phosphate buffer (100 mM, pH 6.0), 16 μl of 0.5% (w/w) H_2_O_2_ solution, 32 μl of 5% (w/v) pyrogallol, plus 10 μl of cell lysates or ddH_2_O (blank control). Absorbance at 420 nm was determined after incubation at 20°C for 3 min. One unit of peroxidase formed 1.0 milligram of purpurogallin from pyrogallol in 20 seconds at pH 6.0 at 20°C, which was equivalent to ~18 μM units per minute at 25°C. The H_2_O_2_ degradation capacity was normalized with total protein level in samples.

### Competition assay *in vivo*

#### Adult mouse model

The streptomycin-treated adult mouse model was used to assess ROS resistance *in vivo* for *cbs* deletion and complement strain, compared with C6706 wild-type, as previously described [7] with the following modifications. Five-week-old CD-1 mice were supplied with drinking water with or without 10 g/L of the antioxidant N-acetyl cysteine (NAC) for one week. Afterwards, 5 g/L streptomycin and 0.05 g/L aspartame were added to the drinking water for the rest of the experiment. One day after streptomycin treatment, mice were administrated with 100 μl of 10% (wt/vol) NaHCO_3_ by gavage, then intragastrically administered with 100 μl of a 1:1 mixture of wild-type and mutant *V*. *cholerae* (approximately 10^9^ CFU for each strain per mouse). Fecal pellets were collected from each mouse at 1, 3, and 5 days post gavage, homogenized by bead beating, and resuspended in PBS buffer, serially diluted, and plated on LB agar containing streptomycin and 5-bromo-4-chloro-3-indolyl-β-D-galactopyranoside (X-gal) for quantification of bacterial loads. Competition index was calculated as the ratio of mutant to wild-type colonies normalized with the input ratio. Competition assay was also conducted for *ΔcbsΔdps* and *Δcbs* in streptomycin-treated adult mouse model to examine the correlation between *cbs*-dependent ROS resistance and iron.

#### Infant mouse model

Five-day-old CD-1 mice were transferred to the 30°C incubator 2 hrs before inoculation. Mice were intragastrically administrated with 50 μl of 1:1 mixture of wild-type and mutant *V*. *cholerae* (approximately 10^6^ CFU for each strain per mouse), then put back into the 30°C incubators. Infant mice were sacrificed 18 hrs post gavage. Samples of the small intestine of each mouse were removed and homogenized in 1.5 ml of PBS buffer, serially diluted, and then plated on LB agar containing streptomycin and X-gal for quantification of bacterial loads. Competition index was calculated as the ratio of mutant to wild-type colonies normalized with the input ratio.

### Proteomic analyses and statistical rationale

#### Sample preparation and LC-MS/MS experiments

The bacterial protein samples were fractionated with 10% SDS-PAGE, and processed into 6 gel bands, followed by in-gel trypsin digestion as previously described [65]. The tryptic peptides were extracted from the gel with 50% acetonitrile (ACN) and 5% formic acid (FA), and vacuum dried. Peptides were resuspended in Solvent A (97% H_2_O, 3% ACN, and 0.1% FA) for proteomic measurements. Liquid chromatography-tandem mass spectrometry (LC-MS/MS) analyses were performed on a hybrid ion trap-Orbitrap mass spectrometer (LTQ-Orbitrap Velos, Thermo Scientific) coupled with nanoflow reversed-phase liquid chromatography (EASY-nLC 1000, Thermo Scientific). The capillary column (75 μm × 150 mm) was home-packed with 4 μm-diameter, 100-Å Magic C18AQ silica-based particles (Michrom BioResources Inc., Auburn, CA). Eluted peptides were electrosprayed directly into the mass spectrometer for MS and MS/MS analyses in a data-dependent acquisition mode. A 45-min gradient was utilized and the percentage of Solvent B (100% ACN and 0.1% FA) was increased from 5% to 35%. The 10 most intense ions from the full MS scan (m/z 350–1,500) were selected for MS/MS analyses. Dynamic exclusion was set with a repeat duration of 24 sec and an exclusion duration of 12 sec. Three independent biological replicates of *V*. *cholerae* samples were analyzed consecutively in 36 LC-MS/MS experiments.

#### Proteomic data analyses

Raw MS files were searched with the MaxQuant software (http://maxquant.org/, Version 1.5.3.30) against the *V*. *cholerae* N16961 protein database, which contained 3,782 protein sequences that were retrieved from the UniProt database under the taxonomic identifier 243277, and label-free quantitation (LFQ) were performed for quantitative analyses. The false-discovery rate (FDR) of peptides and proteins was both controlled at <1%. The peptide assignments that matched the entries in the reverse database or other potential contaminants were filtered with the Perseus software (version 1.5.4.1). Only peptides that were identified in at least 2 biological replicates were used for protein quantification. The imputation of missing values was conducted by using ‘Sequential Knn’ package in R 4.0.3. Two-tailed Student’s *t*-test was carried out to obtain the *p*-values. Proteins with fold changes >1.2 and *p*-values < 0.05 were further compiled and considered as candidates differing between samples.

### Iron content measurement using ICP-MS

#### Bacterial Cultures

Overnight cultures of *V*. *cholerae* strains were 1:100 sub-cultured into 250 ml fresh M9 medium, containing 0.2% casein acid hydrolysate as sole carbon source, and grown at 37°C and 200 rpm until OD_600_ reached around 0.4. Then, 0.5 mM IPTG and 0.02% arabinose were added to induce the expression of *V*. *cholerae cbs* and *dps*, respectively. After 4 hrs’ incubation at 30°C and 200 rpm, an aliquot (1 ml) from each culture were taken for enumeration of bacterial cells, while the rest of cells were collected by high-speed centrifugation and stored immediately at -80°C. Three biological replicates were set for each strain.

#### ICP-MS Analysis

Bacterial cell pellets were frozen, freeze-dried, and homogenized. Samples (~0.1 g) were weighed into polytetrafluoroethylene vessels, with 6 ml of HNO_3_ and 2 ml of 30% H_2_O_2_, for microwave digestion at 185°C, using the ETHOS E microwave digestion system (Milestone, Italy). The digestion solutions were filtered through quantitative filter papers and quantitatively adjusted to 10 ml with ultrapure water. The iron content in samples was measured by ICP-MS analysis of ^57^Fe (Perkin-Elmer, ELAN DRC-e, USA), and normalized with cell numbers.

## Supporting information

S1 Fig Identification of orthologs for H_2_S-producing enzymes in *V*. *cholerae*. (A). CBS orthologs. Left panel, domain architectures of CBS proteins are presented. In CBS proteins, the catalytic core domain PALP (pfam00291) is highly conserved across phyla [28]. There are three PALP-containing proteins in *V*. *cholerae*, encoding by *vc1061*, *vc0968*, and *vc0537*, respectively. Right panel, multiple sequence alignment (MSA) of PALP regions, showing that all the three CBS candidate proteins in *V*. *cholerae* have the active-site loop residues of CBS (outlined with red) [25,27–29]. However, only VC1061 processes the key site for the function of H_2_S biogenesis reported by Devi et al. 2017 [28] (red asterisk). (B). CSE orthologs. Cys/Met metabolism PLP-dependent domain (Cys_Met_Meta_PP, pfam01053) is the conserved domain in CSE proteins. *V*. *cholerae* encodes two Cys_Met_Meta_PP enzymes VC2683 and VC1671. Red and blue asterisks indicate key active-site residues in binging with co-factor pyridoxal-5’-phosphate (PLP) and inhibitor DL-propargylglycine (PAG), respectively [26]. VC1671 is not a CSE homolog, since it lacks four key active-site residues (shaded with yellow). (C). 3MST orthologs. Active-site loop residues of 3MST are shown in red [17]. Hsa, *Homo sapiens*; Dme, *Drosophila melanogaster*; Sce, *Saccharomyces cerevisiae* S288C; Bat, *Bacillus anthracis* str. Sterne; Eco, *Escherichia coli*; Ype, *Yersinia pestis* CO92; Stm, *Salmonella typhimurium* LT2; Vch, *V*. *cholerae*.
(PDF)

S2 Fig H_2_S production in deletion mutants of *V*. *cholerae cbs*, *cse*, and *3mst* candidates. Overnight cultures of single deletion for homologs of *cbs*, *cse*, and *3mst* were 1:100 sub-cultured into fresh Luria–Bertani broth containing 500 μM L-cysteine hydrochloride, followed by stationary incubation at 37°C for 18 hrs. H_2_S production was measured by the darkening of Pb(Ac)_2_ paper stripe during cultivation. The average H_2_S level of the wild-type was set to be 100% for subsequent normalization. Three replicates were sampled for each strain. Significance was determined by *t*-test; *p*-value: ***, <0.001.
(PDF)

S3 Fig *cbs* deletion does not affect biofilm formation, virulence expression, organic hydroperoxide resistance, and growth in *V*. *cholerae*. (A). The biofilm-forming capability of Δ*cbs* at static condition. Overnight cultures of wild-type (WT) or Δ*cbs* were 1:100 sub-cultured into fresh medium. Biofilms formed at solid-liquid interface were analyzed by crystal violet staining after 24 hrs of incubation at 25°C in glass test tubes. (B). *tcpA* expression in Δ*cbs* compared with wild-type (WT). WT and Δ*cbs*, containing promoter-*luxCDABE* transcriptional fusion reporter plasmids of virulence gene *tcpA* were grown aerobically and then 1:100 incubated in LB or AKI medium, at 37°C for 4 hrs without shaking. Luminescence was then measured and normalized against OD_600_. Results are the means and S.D. of three independent experiments. (C). *cbs* expression and the organic hydroperoxide resistance in *V*. *cholerae*. Bacteria were exposed to 100 μM of Cumene hydroperoxide (CHP) for 30 min at their exponential-phase or left untreated, in M9 minimal medium (M9 salts plus 2 mM MgSO_4_, 0.1 mM CaCl_2_, and 0.2% casein acid hydrolysate as sole carbon source) containing 200 μM IPTG. Viability was determined by comparing the CFU in the CHP-challenged and the unchallenged samples. Three replicates were sampled for each strain. No significance was detected (ns). (D). Growth of wild-type (WT/vector), *cbs* deletion mutant (Δ*cbs*/vector), and *cbs*-complemented strain (Δ*cbs*/P_*tac*_*-cbs*) in Luria–Bertani broth or M9 minimal medium (M9 salts plus 2 mM MgSO_4_, 0.1 mM CaCl_2_) with different sole carbon sources. Overnight cultures were 1:1,000 sub-cultured in LB and grown to log-phase at 37°C with shaking. Bacterial cultures were pelleted by centrifugation, washed once, and adjusted OD_600_ to 0.1 with fresh medium. Growth kinetics of test strains were determined by OD_600_ measurement in 96-well microplate using microplate-reader (Spark, Tecan). Samples were incubated at 37°C with shaking, and readings were taken every 20 min for 15 hrs. Four individual experiments were taken.
(PDF)

S4 Fig Identification of key sites for H_2_S production by *V*. *cholerae* CBS. H_2_S production of Δ*cbs* expressing wild-type and point-mutated CBS was examined using Pb(Ac)_2_ paper strips in LB with supplementation of 200 μM L-cysteine hydrochloride. Stained paper strips were scanned and quantified with ImageJ. Average H_2_S level of the Δ*cbs*/vector was set to 100% for subsequent normalization. Three replicates were sampled for each strain. Asterisks indicate statistically significant differences by *t*-test (*, *p*-value < 0.05, **, *p*-value < 0.01, ns, not significant).
(PDF)

S5 Fig Semi-quantification of H_2_S production illustrated in Fig 2. Bacteria were cultured in M9 minimal medium (M9 salts plus 2 mM MgSO_4_, 0.1 mM CaCl_2_, and 0.2% casein acid hydrolysate as sole carbon source) containing appropriate antibiotic and inducers. H_2_S production during growth was monitored with lead acetate paper strips in anaerobic test tubes. Paper strips were scanned for quantification of H_2_S yield, with reference to NaHS standard. The calibration curve for sulfide was Y = 3×10^6^ x—3955.4 (R^2^ = 0.9993), for X representing the amount of sulfide (μmole) and Y representing the integrated density of gray values measured with ImageJ software. Significance was determined by *t*-test; *p*-value: ***, <0.001.
(PDF)

S6 Fig Compared with CBS-derived H_2_S, exogenous H_2_S was less effective in protecting *V*. *cholerae* cells from H_2_O_2_. (A). Survival of Δ*cbs* in M9 minimal medium (M9 salts plus 2 mM MgSO_4_, 0.1 mM CaCl_2_) plus 0.2% casein acid hydrolysate, when NaHS, the rapid donor of H_2_S, was supplied 1hr before under H_2_O_2_ challenge. 1 mM of H_2_O_2_ treatment was performed on log-phase bacteria for 30 min. (B). Semi-quantification of CBS-derived H_2_S production in M9 minimal medium with 0.2% casein acid hydrolysate as the sole carbon source. H_2_S production within 6 hrs was examined with Pb(Ac)_2_ paper strips, and compared with NaHS standards (0, 10 μM, 100 μM, 1 mM). *cbs* expression was induced by 200 μM IPTG.
(PDF)

S7 Fig STRING network analysis of differentially up-regulated proteins in the proteome (S3 Table). (PDF)

S8 Fig H_2_S is the determinant of *cbs* expression promoting catalase activity. Catalase activity in crude extracts of Δ*cbs* strains containing vectors (*cbs*^*-*^*sqr*^*-*^; CBS^-^H_2_S^-^), P_*tac*_*-cbs* (*cbs*^*+*^*sqr*^*-*^; CBS^+^H_2_S^+^) only, or both of P_*tac*_*-cbs* and P_*BAD*_*-sqr* (*cbs*^*+*^*sqr*^*+*^; CBS^+^H_2_S^-^). Cells were grown in M9 minimal medium (M9 salts plus 2 mM MgSO_4_, 0.1 mM CaCl_2_ and 0.2% casein acid hydrolysate), containing 200 μM of IPTG and 0.02% arabinose, and treated with or without H_2_O_2_ (1 mM, 20 min) at their mid-log phase. Three individual experiments were taken. Significance was determined by one-way ANOVA; *p*-value: *, <0.05, **, <0.01, ***, <0.001.
(PDF)

S9 Fig Purification of KatB protein. Hig-tagged *V*. *cholerae* KatB was expressed in M9 minimal medium with 0.2% casein as only carbon source under pBAD promoter in triple-deletion mutant of *cbs*, *katB* and *katG*, with (P_*tac*_*-cbs*) or without (vector) additional *cbs* expression (A). Crude enzyme solution of cells was subjected to CAT activity determination (B), three replicates were sampled for each strain. Significance was determined by *t*-test; *p*-value: **, <0.01.
(PDF)

S10 Fig *cbs* deletion mutant has no defect in colonizing infant mouse. Infant CD1 mice were administrated with wild-type (WT) and Δ*cbs*. Intestines were collected at 18 hrs post inoculation. Bacterial loads were quantified by plating. Competition index (CI) was calculated as the ratio of Δ*cbs* to WT colonies and normalized with the input ratio.
(PDF)

S11 Fig CBS-mediated H_2_S production and cell protection in the absence of exogenous cysteine. Strains were cultured in M9 medium with 0.2% glucose as the sole carbon source. H_2_S production during growth was monitored with lead acetate paper strips, and viability of cells under H_2_O_2_ challenge was examined. The paper strips were scanned for semi-quantification of H_2_S yield with ImageJ and NaHS standard. Significance was determined by *t*-test; *p*-value: *, <0.05; **, <0.01.
(PDF)

S12 Fig CBS-derived H2S helps to preserve the catalase activity of KatG under iron-deficient conditions. Catalase activity in crude extracts of Δ*cbs*Δ*katB*Δ*katG* cells containing *P_BAD_-katG*, and having either *P_tac_-cbs* (*cbs*^+^) or vector control (*cbs*^-^), was examined. Cells were grown in M9 medium containing 0.2% casein acid hydrolysate as the sole carbon source and induced with 0.5 mM IPTG and 0.02% arabinose. Iron chelator 2,2’-bipyridine or FeCl_3_ was added to monitor the iron content of the medium as indicated. Significance was determined by two-way ANOVA from the data of three independent experiments. Significant differences in the mean rank of the catalase activity of each strain at different iron levels are listed in alphabetical order (*cbs*^-^, blue letters; *cbs*^+^, red letters), with the same letter indicating a *p*-value > 0.05. Also indicated are significant differences between *cbs*^-^ and *cbs*^+^ strains at certain 2,2’-bipyridine concentrations; *p*-value, *, <0.05, **, <0.01.
(PDF)

S1 Table Strains and plasmids used in this study. (DOCX)

S2 Table Primers for RT-qPCR in this study. (DOCX)

S3 Table Differentially expressed proteins between *cbs*-deficient and overexpressed *V*. *cholerae* cells. (XLSX)
