# Supplementary material for: CBS-derived H2S facilitates host colonization of Vibrio cholerae by promoting the iron-dependent catalase activity of KatB
Source: PLoS Pathog. 2021 Jul 20;17(7):e1009763. doi: 10.1371/journal.ppat.1009763 (PMC8324212; doi:10.1371/journal.ppat.1009763)
Supplement: S1 Fig — (A). CBS orthologs. Left panel, domain architectures of CBS proteins are presented. In CBS proteins, the catalytic core domain PALP (pfam00291) is highly conserved across phyla [28]. There are three PALP-containing proteins in V. cholerae, encoding by vc1061, vc0968, and vc0537, respectively. Right panel, multiple sequence alignment (MSA) of PALP regions, showing that all the three CBS candidate proteins in V. cholerae have the active-site loop residues of CBS (outlined with red) [25,27–29]. However, only VC1061 processes the key site for the function of H2S biogenesis reported by Devi et al. 2017 [28] (red asterisk). (B). CSE orthologs. Cys/Met metabolism PLP-dependent domain (Cys_Met_Meta_PP, pfam01053) is the conserved domain in CSE proteins. V. cholerae encodes two Cys_Met_Meta_PP enzymes VC2683 and VC1671. Red and blue asterisks indicate key active-site residues in binging with co-factor pyridoxal-5’-phosphate (PLP) and inhibitor DL-propargylglycine (PAG), respectively [26]. VC1671 is not a CSE homolog, since it lacks four key active-site residues (shaded with yellow). (C). 3MST orthologs. Active-site loop residues of 3MST are shown in red [17]. Hsa, Homo sapiens; Dme, Drosophila melanogaster; Sce, Saccharomyces cerevisiae S288C; Bat, Bacillus anthracis str. Sterne; Eco, Escherichia coli; Ype, Yersinia pestis CO92; Stm, Salmonella typhimurium LT2; Vch, V. cholerae. (PDF) [file ppat.1009763.s001.pdf]

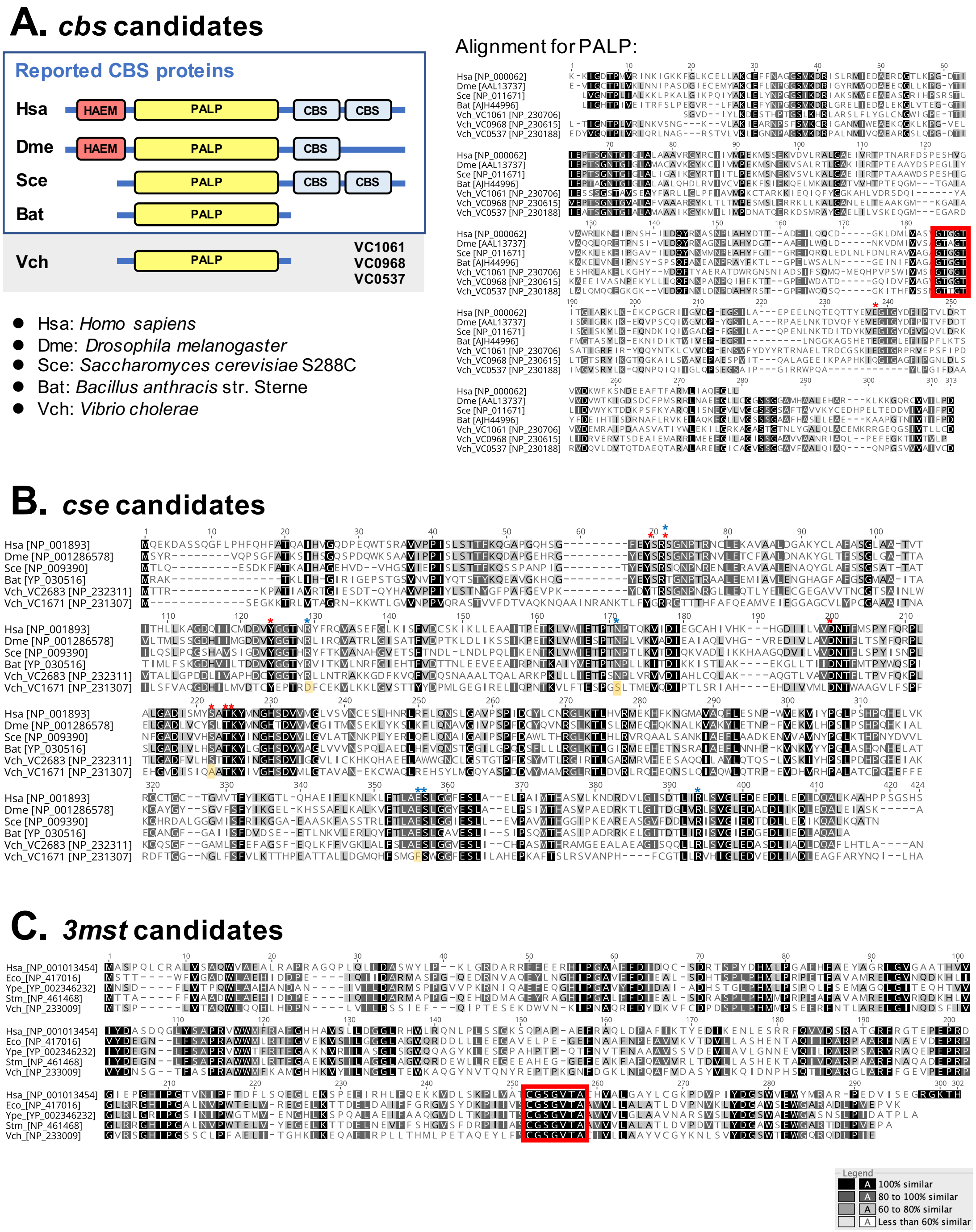

S1 Fig. Identification of orthologs for H_2S-producing enzymes in *V. cholerae*.

(A) CBS orthologs. Left panel, domain architectures of CBS proteins are presented. In CBS proteins, the catalytic core domain PALP (pfam00291) is highly conserved across phyla [1]. There are three PALP-containing proteins in *V. cholerae*, encoding

by *vc1061*, *vc0968*, and *vc0537*, respectively. Right panel, multiple sequence alignment (MSA) of PALP regions, showing that all the three CBS candidate proteins in *V. cholerae* have the active-site loop residues of CBS (outlined with red) [1-4]. However, only VC1061 processes the key site for the function of H_2S biogenesis reported by Devi et al. 2017 [1] (red asterisk).

(B) CSE orthologs. Cys/Met metabolism PLP-dependent domain (Cys_Met_Meta_PP, pfam01053) is the conserved domain in CSE proteins. *V. cholerae* encodes two Cys_Met_Meta_PP enzymes VC2683 and VC1671. Red and blue asterisks indicate key active-site residues in binging with co-factor pyridoxal-5'-phosphate (PLP) and inhibitor DL-propargylglycine (PAG), respectively [5]. VC1671 is not a CSE homolog, since it lacks four key active-site residues (shaded with yellow).

(C) 3MST orthologs. Active-site loop residues of 3MST are shown in red [6]. Hsa, *Homo sapiens*; Dme, *Drosophila melanogaster*; Sce, *Saccharomyces cerevisiae* S288C; Bat, *Bacillus anthracis* str. Sterne; Eco, *Escherichia coli*; Ype, *Yersinia pestis* CO92; Stm, *Salmonella typhimurium* LT2; Vch, *V. cholerae*.

Reference

1. Devi S, Rehman SAA, Tarique KF, Gourinath S. Structural characterization and functional analysis of cystathionine beta-synthase: an enzyme involved in the reverse transsulfuration pathway of Bacillus anthracis. Febs J. 2017;284(22):3862-80. doi: 10.1111/febs.14273. PubMed PMID: WOS:000415877100009.

2. Koutmos M, Kabil O, Smith JL, Banerjee R. Structural basis for substrate activation and regulation by cystathionine beta-synthase (CBS) domains in

cystathionine {beta}-synthase. Proc Natl Acad Sci U S A. 2010;107(49):20958-63. Epub 2010/11/18. doi: 10.1073/pnas.1011448107. PubMed PMID: 21081698; PubMed Central PMCID: PMCPMC3000283.

3. Meier M, Janosik M, Kery V, Kraus JP, Burkhard P. Structure of human cystathionine beta-synthase: a unique pyridoxal 5'-phosphate-dependent heme protein. The EMBO journal. 2001;20(15):3910-6. Epub 2001/08/03. doi: 10.1093/emboj/20.15.3910. PubMed PMID: 11483494; PubMed Central PMCID: PMCPMC149156.

4. Tu Y, Kreinbring CA, Hill M, Liu C, Petsko GA, McCune CD, et al. Crystal Structures of Cystathionine β-Synthase from Saccharomyces cerevisiae: One Enzymatic Step at a Time. Biochemistry. 2018;57(22):3134-45. doi: 10.1021/acs.biochem.8b00092. PMID: 29630349; PMCID: PMC6496941.

5. Sun Q, Collins R, Huang S, Holmberg-Schiavone L, Anand GS, Tan CH, et al. Structural basis for the inhibition mechanism of human cystathionine gamma-lyase, an enzyme responsible for the production of H(2)S. J Biol Chem. 2009;284(5):3076-85. Epub 2008/11/21. doi: 10.1074/jbc.M805459200. PubMed PMID: 19019829.

6. Shatalin K, Shatalina E, Mironov A, Nudler E. H2S: A Universal Defense Against Antibiotics in Bacteria. Science. 2011;334(6058):986-90. doi: 10.1126/science.1209855. PubMed PMID: WOS:000297101800058.
