# Supplementary material for: CBS-derived H2S facilitates host colonization of Vibrio cholerae by promoting the iron-dependent catalase activity of KatB
Source: PLoS Pathog. 2021 Jul 20;17(7):e1009763. doi: 10.1371/journal.ppat.1009763 (PMC8324212; doi:10.1371/journal.ppat.1009763)
Supplement: S2 Fig — Overnight cultures of single deletion for homologs of cbs, cse, and 3mst were 1:100 sub-cultured into fresh Luria–Bertani broth containing 500 μM L-cysteine hydrochloride, followed by stationary incubation at 37°C for 18 hrs. H2S production was measured by the darkening of Pb(Ac)2 paper stripe during cultivation. The average H2S level of the wild-type was set to be 100% for subsequent normalization. Three replicates were sampled for each strain. Significance was determined by t-test; p-value: ***, <0.001. (PDF) [file ppat.1009763.s002.pdf]

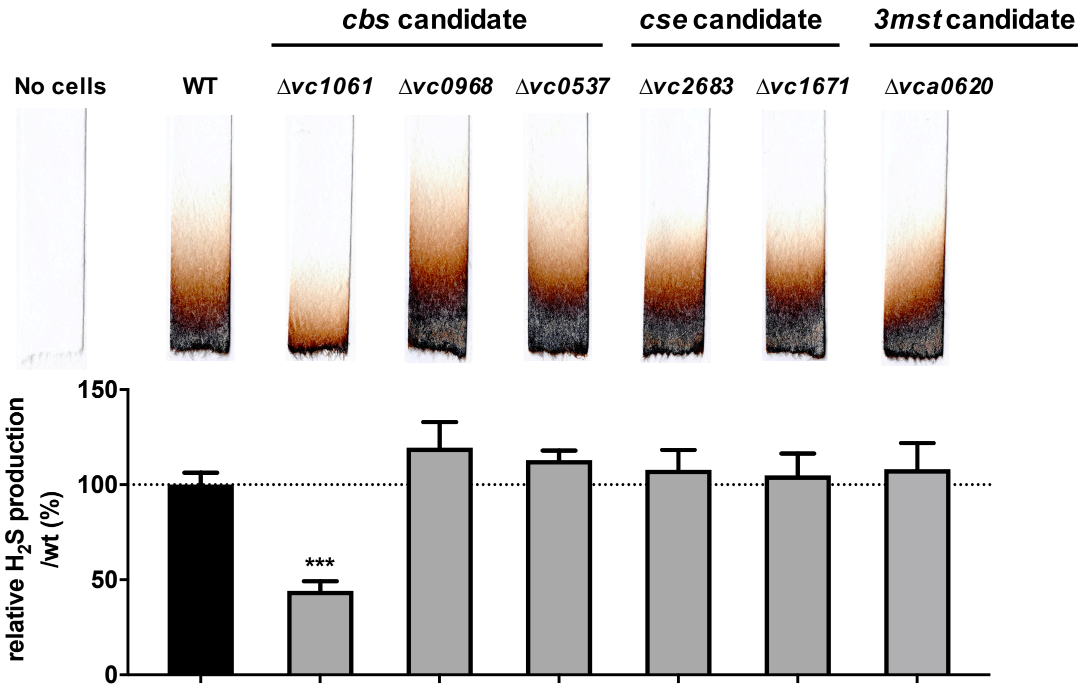

S2 Fig. H_2S production in deletion mutants of *V. cholerae cbs*, *cse*, and *3mst* candidates.

Overnight cultures of single deletion for homologs of *cbs*, *cse*, and *3mst* were 1:100 sub-cultured into fresh Luria–Bertani broth containing 500 μM L-cysteine hydrochloride, followed by stationary incubation at 37°C for 18 hrs. H_2S production was measured by the darkening of $Pb(Ac)_2$ paper stripe during cultivation. The average H_2S level of the wild-type was set to be 100% for subsequent normalization. Three replicates were sampled for each strain. Significance was determined by *t*-test; *p*-value: ***, <0.001.
