# Supplementary material for: CBS-derived H2S facilitates host colonization of Vibrio cholerae by promoting the iron-dependent catalase activity of KatB
Source: PLoS Pathog. 2021 Jul 20;17(7):e1009763. doi: 10.1371/journal.ppat.1009763 (PMC8324212; doi:10.1371/journal.ppat.1009763)
Supplement: S3 Fig — (A). The biofilm-forming capability of Δcbs at static condition. Overnight cultures of wild-type (WT) or Δcbs were 1:100 sub-cultured into fresh medium. Biofilms formed at solid-liquid interface were analyzed by crystal violet staining after 24 hrs of incubation at 25°C in glass test tubes. (B). tcpA expression in Δcbs compared with wild-type (WT). WT and Δcbs, containing promoter-luxCDABE transcriptional fusion reporter plasmids of virulence gene tcpA were grown aerobically and then 1:100 incubated in LB or AKI medium, at 37°C for 4 hrs without shaking. Luminescence was then measured and normalized against OD600. Results are the means and S.D. of three independent experiments. (C). cbs expression and the organic hydroperoxide resistance in V. cholerae. Bacteria were exposed to 100 μM of Cumene hydroperoxide (CHP) for 30 min at their exponential-phase or left untreated, in M9 minimal medium (M9 salts plus 2 mM MgSO4, 0.1 mM CaCl2, and 0.2% casein acid hydrolysate as sole carbon source) containing 200 μM IPTG. Viability was determined by comparing the CFU in the CHP-challenged and the unchallenged samples. Three replicates were sampled for each strain. No significance was detected (ns). (D). Growth of wild-type (WT/vector), cbs deletion mutant (Δcbs/vector), and cbs-complemented strain (Δcbs/Ptac-cbs) in Luria–Bertani broth or M9 minimal medium (M9 salts plus 2 mM MgSO4, 0.1 mM CaCl2) with different sole carbon sources. Overnight cultures were 1:1,000 sub-cultured in LB and grown to log-phase at 37°C with shaking. Bacterial cultures were pelleted by centrifugation, washed once, and adjusted OD600 to 0.1 with fresh medium. Growth kinetics of test strains were determined by OD600 measurement in 96-well microplate using microplate-reader (Spark, Tecan). Samples were incubated at 37°C with shaking, and readings were taken every 20 min for 15 hrs. Four individual experiments were taken. (PDF) [file ppat.1009763.s003.pdf]

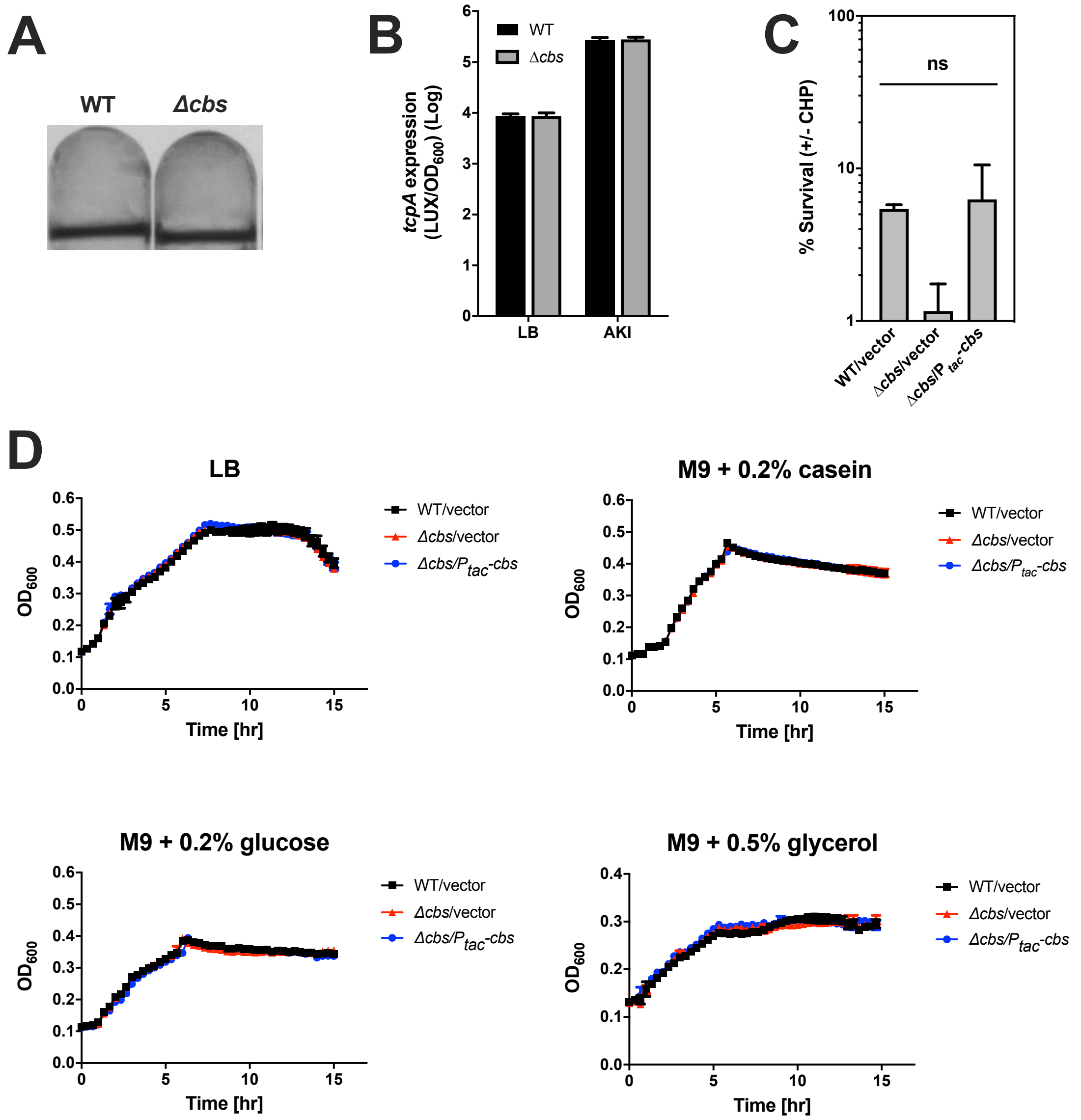

S3 Fig. *cbs* deletion does not affect biofilm formation, virulence expression, organic hydroperoxide resistance, and growth in *V. cholerae*.

(A) The biofilm-forming capability of Δ*cbs* at static condition. Overnight cultures of wild-type (WT) or Δ*cbs* were 1:100 sub-cultured into fresh medium. Biofilms formed at solid-liquid interface were analyzed by crystal violet staining after 24 hrs of incubation at 25°C in glass test tubes.

(B) *tcpA* expression in Δ*cbs* compared with wild-type (WT). WT and Δ*cbs*, containing

promoter-*luxCDABE* transcriptional fusion reporter plasmids of virulence gene *tcpA* were grown aerobically and then 1:100 incubated in LB or AKI medium, at 37°C for 4 hrs without shaking. Luminescence was then measured and normalized against OD_{600}. Results are the means and S.D. of three independent experiments.

(C) *cbs* expression and the organic hydroperoxide resistance in *V. cholerae*. Bacteria were exposed to 100 μM of Cumene hydroperoxide (CHP) for 30 min at their exponential-phase or left untreated, in M9 minimal medium (M9 salts plus 2 mM $MgSO_4$, 0.1 mM $CaCl_2$, and 0.2% casein acid hydrolysate as sole carbon source) containing 200 μM IPTG. Viability was determined by comparing the CFU in the CHP-challenged and the unchallenged samples. Three replicates were sampled for each strain. No significance was detected (ns).

(D) Growth of wild-type (WT/vector), *cbs* deletion mutant (Δ*cbs*/vector), and *cbs*-complemented strain (Δ*cbs*/P_{tac}-*cbs*) in Luria–Bertani broth or M9 minimal medium (M9 salts plus 2 mM $MgSO_4$, 0.1 mM $CaCl_2$) with different sole carbon sources. Overnight cultures were 1:1,000 sub-cultured in LB and grown to log-phase at 37°C with shaking. Bacterial cultures were pelleted by centrifugation, washed once, and adjusted OD_{600} to 0.1 with fresh medium. Growth kinetics of test strains were determined by OD_{600} measurement in 96-well microplate using microplate-reader (Spark, Tecan). Samples were incubated at 37°C with shaking, and readings were taken every 20 min for 15 hrs. Four individual experiments were taken.
