# Supplementary material for: CBS-derived H2S facilitates host colonization of Vibrio cholerae by promoting the iron-dependent catalase activity of KatB
Source: PLoS Pathog. 2021 Jul 20;17(7):e1009763. doi: 10.1371/journal.ppat.1009763 (PMC8324212; doi:10.1371/journal.ppat.1009763)
Supplement: S4 Fig — H2S production of Δcbs expressing wild-type and point-mutated CBS was examined using Pb(Ac)2 paper strips in LB with supplementation of 200 μM L-cysteine hydrochloride. Stained paper strips were scanned and quantified with ImageJ. Average H2S level of the Δcbs/vector was set to 100% for subsequent normalization. Three replicates were sampled for each strain. Asterisks indicate statistically significant differences by t-test (*, p-value < 0.05, **, p-value < 0.01, ns, not significant). (PDF) [file ppat.1009763.s004.pdf]

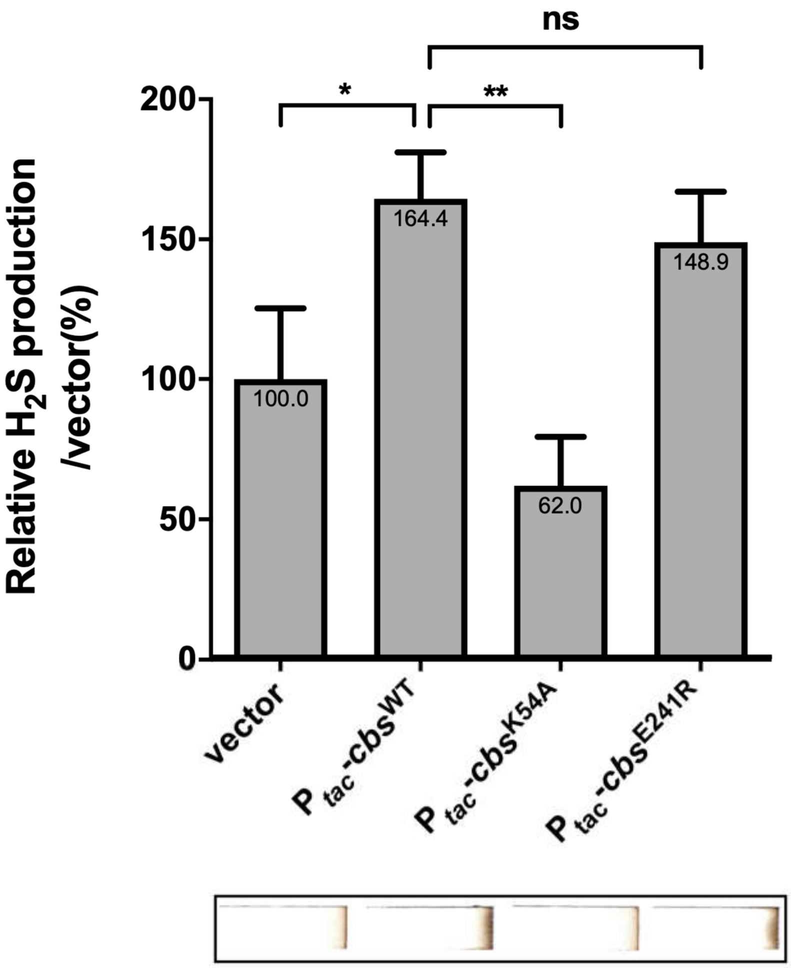

S4 Fig. Identification of key sites for H_2S production by *V. cholerae* CBS.

H_2S production of Δ*cbs* expressing wild-type and point-mutated CBS was examined using $Pb(Ac)_2$ paper strips in LB with supplementation of 200 μM L-cysteine hydrochloride. Stained paper strips were scanned and quantified with ImageJ. Average H_2S level of the Δ*cbs*/vector was set to 100% for subsequent normalization. Three replicates were sampled for each strain. Asterisks indicate statistically significant differences by *t*-test (*, *p*-value < 0.05, **, *p*-value < 0.01, ns, not significant).
