# Supplementary material for: CBS-derived H2S facilitates host colonization of Vibrio cholerae by promoting the iron-dependent catalase activity of KatB
Source: PLoS Pathog. 2021 Jul 20;17(7):e1009763. doi: 10.1371/journal.ppat.1009763 (PMC8324212; doi:10.1371/journal.ppat.1009763)
Supplement: S5 Fig — Bacteria were cultured in M9 minimal medium (M9 salts plus 2 mM MgSO4, 0.1 mM CaCl2, and 0.2% casein acid hydrolysate as sole carbon source) containing appropriate antibiotic and inducers. H2S production during growth was monitored with lead acetate paper strips in anaerobic test tubes. Paper strips were scanned for quantification of H2S yield, with reference to NaHS standard. The calibration curve for sulfide was Y = 3×106 x—3955.4 (R2 = 0.9993), for X representing the amount of sulfide (μmole) and Y representing the integrated density of gray values measured with ImageJ software. Significance was determined by t-test; p-value: ***, <0.001. (PDF) [file ppat.1009763.s005.pdf]

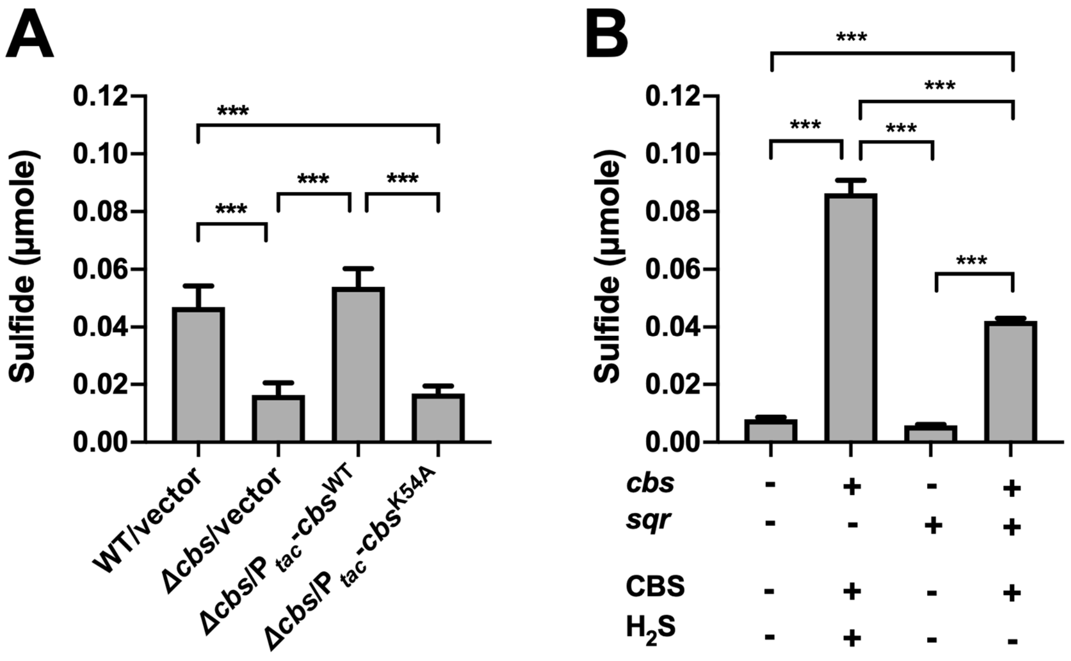

S5 Fig. Semi-quantification of H_2S production illustrated in Fig 2.

Bacteria were cultured in M9 minimal medium (M9 salts plus 2 mM $MgSO_4$, 0.1 mM $CaCl_2$, and 0.2% casein acid hydrolysate as sole carbon source) containing appropriate antibiotic and inducers. H_2S production during growth was monitored with lead acetate paper strips in anaerobic test tubes. Paper strips were scanned for quantification of H_2S yield, with reference to NaHS standard. The calibration curve for sulfide was $Y= 3\times10^6 x - 3955.4$ (R^2=0.9993), for X representing the amount of sulfide (μmole) and Y representing the integrated density of gray values measured with ImageJ software. Significance was determined by *t*-test; *p*-value: ***, <0.001.
