# Supplementary material for: CBS-derived H2S facilitates host colonization of Vibrio cholerae by promoting the iron-dependent catalase activity of KatB
Source: PLoS Pathog. 2021 Jul 20;17(7):e1009763. doi: 10.1371/journal.ppat.1009763 (PMC8324212; doi:10.1371/journal.ppat.1009763)
Supplement: S6 Fig — (A). Survival of Δcbs in M9 minimal medium (M9 salts plus 2 mM MgSO4, 0.1 mM CaCl2) plus 0.2% casein acid hydrolysate, when NaHS, the rapid donor of H2S, was supplied 1hr before under H2O2 challenge. 1 mM of H2O2 treatment was performed on log-phase bacteria for 30 min. (B). Semi-quantification of CBS-derived H2S production in M9 minimal medium with 0.2% casein acid hydrolysate as the sole carbon source. H2S production within 6 hrs was examined with Pb(Ac)2 paper strips, and compared with NaHS standards (0, 10 μM, 100 μM, 1 mM). cbs expression was induced by 200 μM IPTG. (PDF) [file ppat.1009763.s006.pdf]

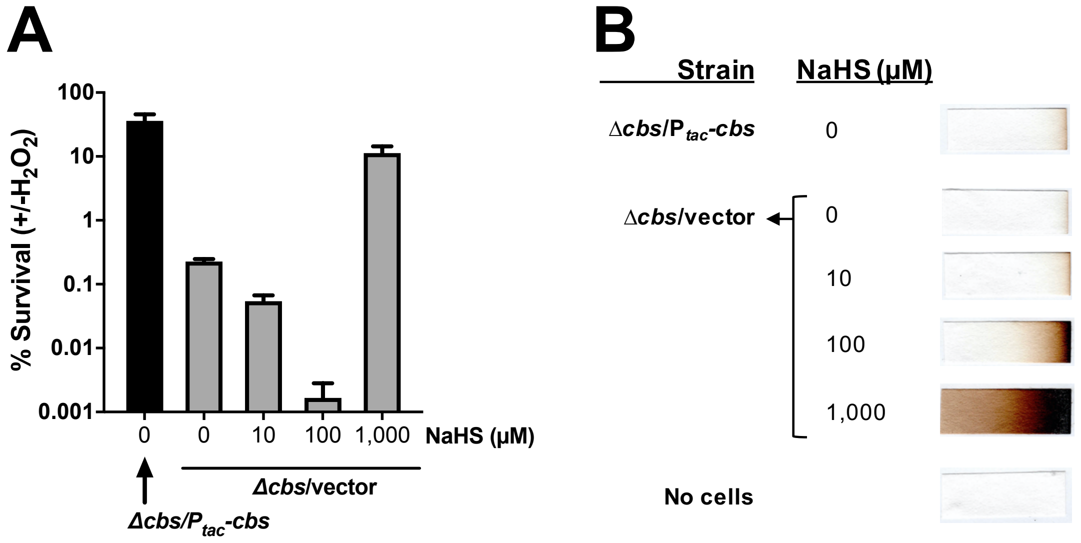

S6 Fig. Compared with CBS-derived H_2S, exogenous H_2S was less effective in protecting *V. cholerae* cells from H_2O_2.

(A) Survival of Δ*cbs* in M9 minimal medium (M9 salts plus 2 mM $MgSO_4$, 0.1 mM $CaCl_2$) plus 0.2% casein acid hydrolysate, when NaHS, the rapid donor of H_2S, was supplied 1hr before under H_2O_2 challenge. 1 mM of H_2O_2 treatment was performed on log-phase bacteria for 30 min.

(B) Semi-quantification of CBS-derived H_2S production in M9 minimal medium with 0.2% casein acid hydrolysate as the sole carbon source. H_2S production within 6 hrs was examined with $Pb(Ac)_2$ paper strips, and compared with NaHS standards (0, 10 μM, 100 μM, 1 mM). *cbs* expression was induced by 200 μM IPTG.
