# Supplementary material for: CBS-derived H2S facilitates host colonization of Vibrio cholerae by promoting the iron-dependent catalase activity of KatB
Source: PLoS Pathog. 2021 Jul 20;17(7):e1009763. doi: 10.1371/journal.ppat.1009763 (PMC8324212; doi:10.1371/journal.ppat.1009763)
Supplement: S8 Fig — Catalase activity in crude extracts of Δcbs strains containing vectors (cbs-sqr-; CBS-H2S-), Ptac-cbs (cbs+sqr-; CBS+H2S+) only, or both of Ptac-cbs and PBAD-sqr (cbs+sqr+; CBS+H2S-). Cells were grown in M9 minimal medium (M9 salts plus 2 mM MgSO4, 0.1 mM CaCl2 and 0.2% casein acid hydrolysate), containing 200 μM of IPTG and 0.02% arabinose, and treated with or without H2O2 (1 mM, 20 min) at their mid-log phase. Three individual experiments were taken. Significance was determined by one-way ANOVA; p-value: *, <0.05, **, <0.01, ***, <0.001. (PDF) [file ppat.1009763.s008.pdf]

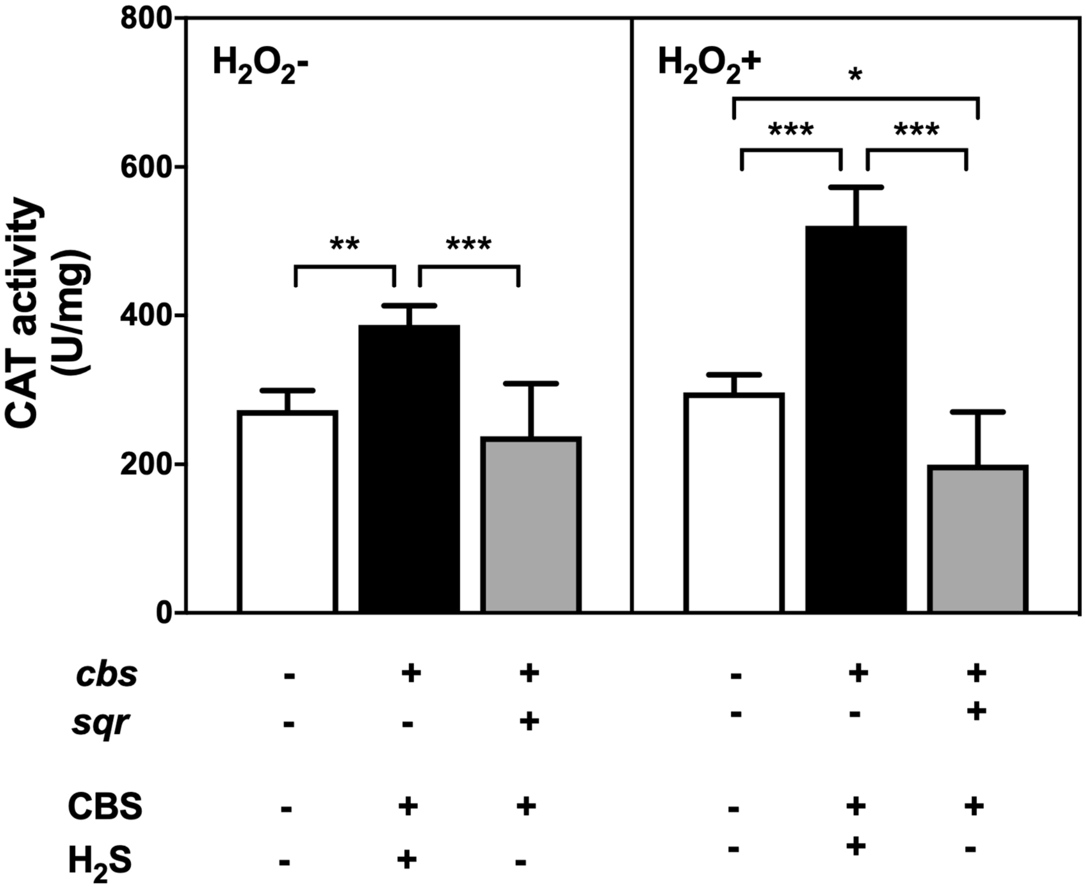

S8 Fig. H_2S is the determinant of *cbs* expression promoting catalase activity.

Catalase activity in crude extracts of Δ*cbs* strains containing vectors (*cbs*$^-$*sqr*$^-$; CBS$^-$ H_2S^-), P$_{tac}$-*cbs* (*cbs*$^+$*sqr*$^-$; CBS$^+$$H_2S^+$) only, or both of P$_{tac}$-*cbs* and P$_{BAD}$-*sqr* (*cbs*$^+$*sqr*$^+$; CBS$^+$$H_2S^-$). Cells were grown in M9 minimal medium (M9 salts plus 2 mM $MgSO_4$, 0.1 mM $CaCl_2$ and 0.2% casein acid hydrolysate), containing 200 µM of IPTG and 0.02% arabinose, and treated with or without H_2O_2 (1 mM, 20 min) at their mid-log phase. Three individual experiments were taken. Significance was determined by one-way ANOVA; *p*-value: *, <0.05, **, <0.01, ***, <0.001.
