# Supplementary material for: CBS-derived H2S facilitates host colonization of Vibrio cholerae by promoting the iron-dependent catalase activity of KatB
Source: PLoS Pathog. 2021 Jul 20;17(7):e1009763. doi: 10.1371/journal.ppat.1009763 (PMC8324212; doi:10.1371/journal.ppat.1009763)
Supplement: S9 Fig — Hig-tagged V. cholerae KatB was expressed in M9 minimal medium with 0.2% casein as only carbon source under pBAD promoter in triple-deletion mutant of cbs, katB and katG, with (Ptac-cbs) or without (vector) additional cbs expression (A). Crude enzyme solution of cells was subjected to CAT activity determination (B), three replicates were sampled for each strain. Significance was determined by t-test; p-value: **, <0.01. (PDF) [file ppat.1009763.s009.pdf]

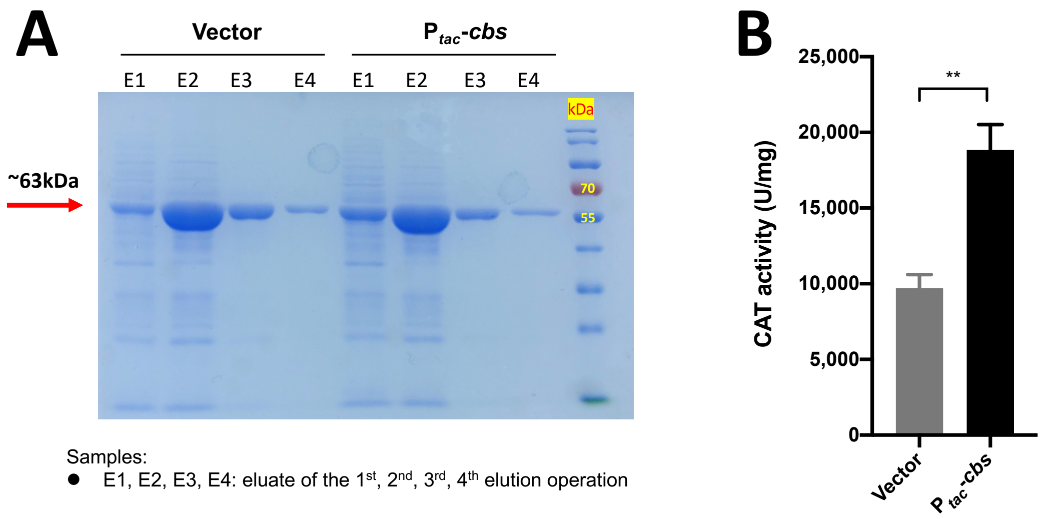

S9 Fig. Purification of KatB protein.

Hig-tagged *V. cholerae* KatB was expressed in M9 minimal medium with 0.2% casein as only carbon source under pBAD promoter in triple-deletion mutant of *cbs*, *katB* and *katG*, with (P_{tac}-*cbs*) or without (vector) additional *cbs* expression (A). Crude enzyme solution of cells was subjected to CAT activity determination (B), three replicates were sampled for each strain. Significance was determined by *t*-test; *p*-value: **, <0.01.
