# Supplementary material for: CBS-derived H2S facilitates host colonization of Vibrio cholerae by promoting the iron-dependent catalase activity of KatB
Source: PLoS Pathog. 2021 Jul 20;17(7):e1009763. doi: 10.1371/journal.ppat.1009763 (PMC8324212; doi:10.1371/journal.ppat.1009763)
Supplement: S10 Fig — Infant CD1 mice were administrated with wild-type (WT) and Δcbs. Intestines were collected at 18 hrs post inoculation. Bacterial loads were quantified by plating. Competition index (CI) was calculated as the ratio of Δcbs to WT colonies and normalized with the input ratio. (PDF) [file ppat.1009763.s010.pdf]

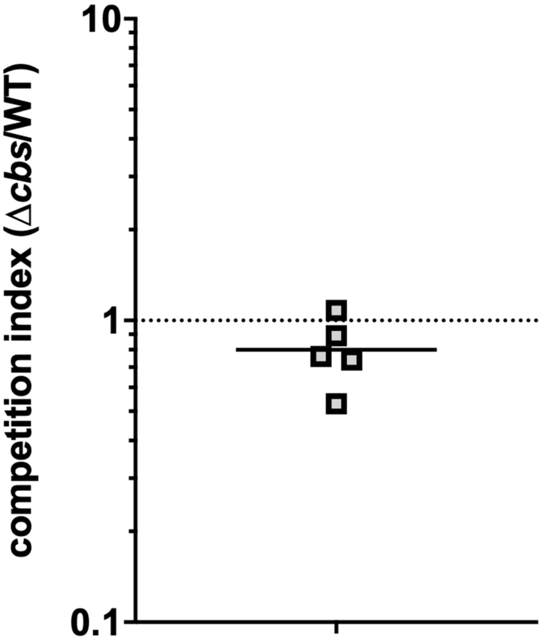

S10 Fig. ***cbs*** **deletion mutant has no defect in colonizing infant mouse.**

Infant CD1 mice were administrated with wild-type (WT) and Δ*cbs*. Intestines were collected at 18 hrs post inoculation. Bacterial loads were quantified by plating. Competition index (CI) was calculated as the ration of Δ*cbs* to WT colonies and normalized with the input ratio.
