# Supplementary material for: CBS-derived H2S facilitates host colonization of Vibrio cholerae by promoting the iron-dependent catalase activity of KatB
Source: PLoS Pathog. 2021 Jul 20;17(7):e1009763. doi: 10.1371/journal.ppat.1009763 (PMC8324212; doi:10.1371/journal.ppat.1009763)
Supplement: S11 Fig — Strains were cultured in M9 medium with 0.2% glucose as the sole carbon source. H2S production during growth was monitored with lead acetate paper strips, and viability of cells under H2O2 challenge was examined. The paper strips were scanned for semi-quantification of H2S yield with ImageJ and NaHS standard. Significance was determined by t-test; p-value: *, <0.05; **, <0.01. (PDF) [file ppat.1009763.s011.pdf]

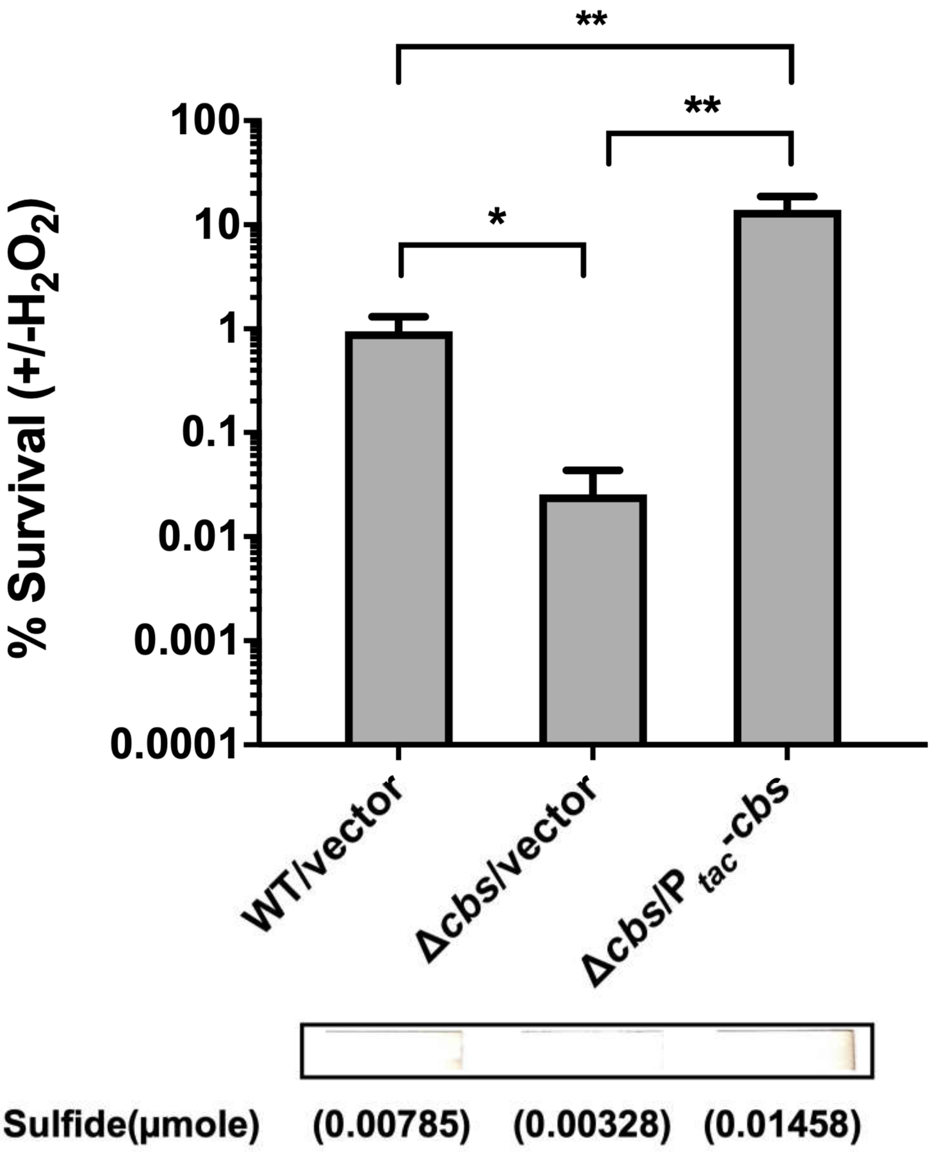

S11 Fig. CBS-mediated H_2S production and cell protection in the absence of exogenous cysteine.

Strains were cultured in M9 medium with 0.2% glucose as the sole carbon source. H_2S production during growth was monitored with lead acetate paper strips, and viability of cells under H_2O_2 challenge was examined. The paper strips were scanned for semi-quantification of H_2S yield with ImageJ and NaHS standard. Significance was determined by *t*-test; *p*-value: *, <0.05; **, <0.01.
