# Supplementary material for: CBS-derived H2S facilitates host colonization of Vibrio cholerae by promoting the iron-dependent catalase activity of KatB
Source: PLoS Pathog. 2021 Jul 20;17(7):e1009763. doi: 10.1371/journal.ppat.1009763 (PMC8324212; doi:10.1371/journal.ppat.1009763)
Supplement: S12 Fig — Catalase activity in crude extracts of ΔcbsΔkatBΔkatG cells containing PBAD-katG, and having either Ptac-cbs (cbs+) or vector control (cbs-), was examined. Cells were grown in M9 medium containing 0.2% casein acid hydrolysate as the sole carbon source and induced with 0.5 mM IPTG and 0.02% arabinose. Iron chelator 2,2’-bipyridine or FeCl3 was added to monitor the iron content of the medium as indicated. Significance was determined by two-way ANOVA from the data of three independent experiments. Significant differences in the mean rank of the catalase activity of each strain at different iron levels are listed in alphabetical order (cbs-, blue letters; cbs+, red letters), with the same letter indicating a p-value > 0.05. Also indicated are significant differences between cbs- and cbs+ strains at certain 2,2’-bipyridine concentrations; p-value, *, <0.05, **, <0.01. (PDF) [file ppat.1009763.s012.pdf]

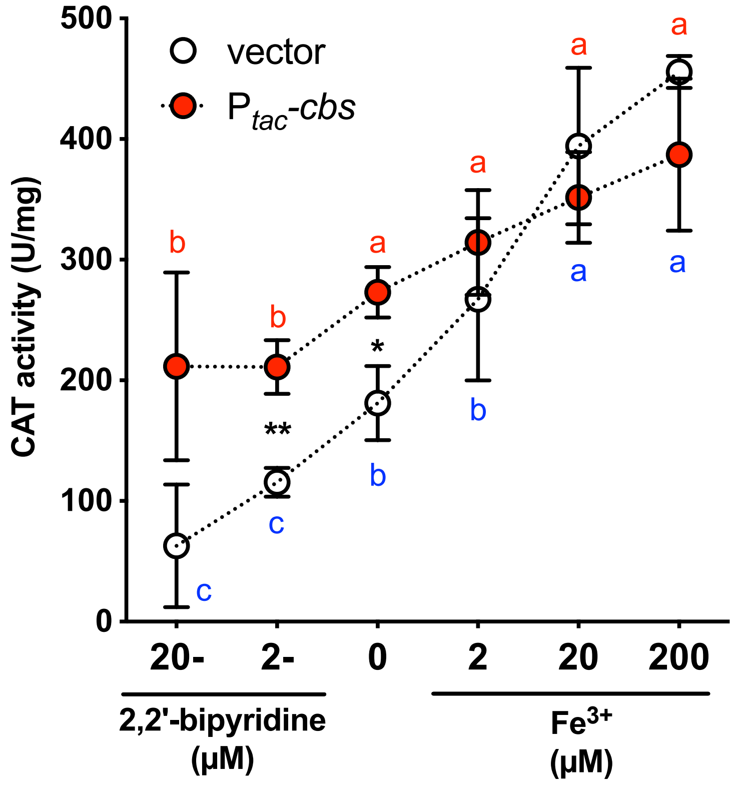

S12 Fig. CBS-derived H_2S helps to preserve the catalase activity of KatG under iron-deficient conditions.

Catalase activity in crude extracts of Δ*cbs*Δ*katB*Δ*katG* cells containing P_{BAD}-*katG*, and having either P_{tac}-*cbs* (*cbs*$^+$) or vector control (*cbs*$^-$), was examined. Cells were grown in M9 medium containing 0.2% casein acid hydrolysate as the sole carbon source and induced with 0.5 mM IPTG and 0.02% arabinose. Iron chelator 2,2'-bipyridine or $FeCl_3$ was added to monitor the iron content of the medium as indicated. Significance was determined by two-way ANOVA from the data of three independent experiments. Significant differences in the mean rank of the catalase activity of each strain at different iron levels are listed in alphabetical order (*cbs*$^-$, blue letters; *cbs*$^+$, red letters), with the same letter indicating a *p*-value > 0.05. Also indicated are

significant differences between *cbs*$^{-}$ and *cbs*$^{+}$ strains at certain 2,2'-bipyridine concentrations; *p*-value, *, <0.05, **, <0.01.
