# Supplementary material for: CBS-derived H2S facilitates host colonization of Vibrio cholerae by promoting the iron-dependent catalase activity of KatB
Source: PLoS Pathog. 2021 Jul 20;17(7):e1009763. doi: 10.1371/journal.ppat.1009763 (PMC8324212; doi:10.1371/journal.ppat.1009763)
Supplement: S1 Table — (DOCX) [file ppat.1009763.s013.docx]

**S1 Table. Strains and plasmids used in this study.**

| **Strain or plasmid** | **Genotype or description** | **Reference or source** |
| --- | --- | --- |
| **Strains** |  |  |
| ***E. coli*** |  |  |
| Sm10 λpir | *thi thr leu tonA lacY supE recA*::RP4-2-Tc::Mu (λ pirR6K) Kan^r^ | ^[1]^ |
| DH5α λpir | *supE44* Δ*lac*U169 (Φ*lacZ*ΔM15) *recA1 endA1 hsdR17 thi-1 gyrA96 relA1 λpir* | ^[2]^ |
| DH5α | Φ80d*lacZ*ΔM15 *recA1 endA1 gyrA96* *thi-1 hsdR17 supE44 relA1* Δ(*lacZYA-argF*)*U169* | ^[2]^ |
| ***V. cholerae*** |  |  |
| C6706 | *Vibrio cholerae* O1 El Tor C6706, wild type, Str^r^ | ^[3]^ |
| C6706 *lacZ*- | C6706 *lacZ* deletion Str^r^ | ^[4]^ |
| YMV8 | C6706 Δ*cbs* (VC1061) Str^r^ | This study |
| YMV16 | C6706 Δ*cse* (VC2683) Str^r^ | This study |
| YMV17 | C6706 Δ*3mst* (VCA0620) Str^r^ | This study |
| YMV18 | C6706 Δ*cbs* Δ*cse* Str^r^ | This study |
| YMV19 | C6706 Δ*cse* Δ*3mst* Str^r^ | This study |
| YMV25 | C6706 Δ*cbs* Δ*cse* Δ*3mst* Str^r^ | This study |
| YMV26 | C6706 Δ*vc0968* Str^r^ | This study |
| YMV24 | C6706 Δ*vc0537* Str^r^ | This study |
| YMV20 | C6706 Δ*vc1671* Str^r^ | This study |
| YMV13 | C6706 Δ*vc0384-5* (i.e. Δ*cysI*Δ*cysJ*) Str^rs^ | This study |
| YMV41 | C6706 Δ*cbs* *lacZ::P_lac_-cbs* Str^r^ | This study |
| YMV40 | C6706 Δ*katB* (VC1585) Δ*katG* (VC1560) Str^r^ | This study |
| YMV43 | C6706 Δ*katB* Δ*katG* Δ*cbs* Str^r^ | This study |
| YMV44 | C6706 Δ*dps* Str^r^ | This study |
| YMV45 | C6706 Δ*cbs* Δ*dps* Str^r^ | This study |
| **Plasmids** |  |  |
| pWM91 | Suicide vector for allelic exchange, *mob*^+^, Amp^r^ | ^[5]^ |
| pMal-c2x | Plasmid containing an IPTG-inducible P*_tac_* promoter, Amp^r^, ColE1 origin of replication | New England Biolabs |
| pYM22 | pMa-c2x with ORF of *cbs* instead of *malE* gene, Amp^r^ | This study |
| pJL1 | Suicide vector for allele exchange in *V. cholerae* *lacZ*, *mob*^+^, Amp^r^ | ^[6]^ |
| pYM34 | pJL1 with P*_lac_*-*cbs* inserted in the NotI site, Amp^r^ | This study |
| pACYC177 | Low copy number plasmid vector, Amp^r^ Kan^r^, p15A origin of replication | ^[7]^ |
| pYM24 | pACYC177 with P*_BAD_* promoter inserted at the *bla* gene, Kan^r^ | This study |
| pYM40 | pYM24-*katB*, Kan^r^ | This study |
| pYM41 | pYM24-*katG*, Kan^r^ | This study |
| pYM42 | pYM24 with ORF of *Staphylococcus aureus sqr* gene fused to P*_BAD_* promoter, Kan^r^ | This study |
| pYM49 | pYM24-*katB*-his6, Kan^r^ | This study |
| pYM50 | pYM24-*dps*, Kan^r^ | This study |

**References**

1. Miller VL, Mekalanos JJ. A NOVEL SUICIDE VECTOR AND ITS USE IN CONSTRUCTION OF INSERTION MUTATIONS - OSMOREGULATION OF OUTER-MEMBRANE PROTEINS AND VIRULENCE DETERMINANTS IN VIBRIO-CHOLERAE REQUIRES TOXR. J Bacteriol. 1988;170(6):2575-83. doi: 10.1128/jb.170.6.2575-2583.1988. PubMed PMID: WOS:A1988N699900025.

2. Hanahan D. STUDIES ON TRANSFORMATION OF ESCHERICHIA-COLI WITH PLASMIDS. J Mol Biol. 1983;166(4):557-80. doi: 10.1016/s0022-2836(83)80284-8. PubMed PMID: WOS:A1983QW37900004.

3. Joelsson A, Liu Z, Zhu J. Genetic and phenotypic diversity of quorum-sensing systems in clinical and environmental isolates of Vibrio cholerae. Infect Immun. 2006;74(2):1141-7. Epub 2006/01/24. doi: 10.1128/iai.74.2.1141-1147.2006. PubMed PMID: 16428762; PubMed Central PMCID: PMCPMC1360356.

4. Zhu J, Miller MB, Vance RE, Dziejman M, Bassler BL, Mekalanos JJ. Quorum-sensing regulators control virulence gene expression in Vibrio cholerae. Proceedings of the National Academy of Sciences. 2002;99(5):3129-34. doi: 10.1073/pnas.052694299.

5. Metcalf WW, Jiang W, Daniels LL, Kim SK, Haldimann A, Wanner BL. Conditionally replicative and conjugative plasmids carrying lacZ alpha for cloning, mutagenesis, and allele replacement in bacteria. Plasmid. 1996;35(1):1-13. Epub 1996/01/01. doi: 10.1006/plas.1996.0001. PubMed PMID: 8693022.

6. Liu Z, Yang MH, Peterfreund GL, Tsou AM, Selamoglu N, Daldal F, et al. Vibrio cholerae anaerobic induction of virulence gene expression is controlled by thiol-based switches of virulence regulator AphB. Proc Natl Acad Sci U S A. 2011;108(2):810-5. doi: 10.1073/pnas.1014640108. PubMed PMID: WOS:000286097700070.

7. Chang AC, Cohen SN. Construction and characterization of amplifiable multicopy DNA cloning vehicles derived from the P15A cryptic miniplasmid. J Bacteriol. 1978;134(3):1141-56.
