# Supplementary material for: CBS-derived H2S facilitates host colonization of Vibrio cholerae by promoting the iron-dependent catalase activity of KatB
Source: PLoS Pathog. 2021 Jul 20;17(7):e1009763. doi: 10.1371/journal.ppat.1009763 (PMC8324212; doi:10.1371/journal.ppat.1009763)
Supplement: S2 Table — (DOCX) [file ppat.1009763.s014.docx]

**S2 Table. Primers for RT-qPCR in this study.**

| \| **target gene** \| **primer name** \| **sequence** \| \| --- \| --- \| --- \| \| *16S rRNA* \| 16S-RT-F \| 5' CGGTAATACGGAGGGTGCAA 3' \| \|  \| 16S-RT-R \| 5' CACCTGCATGCGCTTTACG 3' \| \| *katB* (VC1585) \| katB-RT-F \| 5' ATTTGCGTATGCCGATACAC 3' \| \|  \| katB-RT-R \| 5' GACTGGGTTGGTAGTTAATGTC 3' \| \| *katG* (VC1560) \| katG-RT-F \| 5' GGTAAAGTTCTGGCGGTATTAG 3' \| \|  \| katG-RT-R \| 5' GACTCAACATCGGTTTGTTC 3' \| \| *ahpC* (VC0731) \| ahpC-RT-F \| 5' TCACAACGCATGGCGTAAC 3' \| \|  \| ahpC-RT-R \| 5' CTTCGTCGATCAGGAATGAACC 3' \| \| *prxA* (VC2637) \| prxA-RT-F \| 5' CTTGGGTCAATGTGACTAGC 3' \| \|  \| prxA-RT-R \| 5' ACCGATACGCACAGAATG 3' \| \| *ohrA* (VCA1006) \| ohrA-RT-F \| 5' AACGCGATTCTGCACGTC 3' \| \|  \| ohrA-RT-R \| 5' CAACGGTCACCAACTGTCTG 3' \| \| *sodA* (VC2694) \| sodA-RT-F \| 5' CATGGAGGTGCATTACAG 3' \| \|  \| sodA-RT-R \| 5' CAGCGTTAGGCTTCATAC 3' \| \| *sodB* (VC2045) \| sodB-RT-F \| 5' AAAGCTGACGGCACTCTG 3' \| \|  \| sodB-RT-R \| 5' GATGTAGTACGCGTGTTCC 3' \| \| *sodC* (VC1583) \| sodC-RT-F \| 5' CCGTAACTGATAGCGAGTATG 3' \| \|  \| sodC-RT-R \| 5' TCTTTGGACGAGGTTTCAC 3' \| |  |
| --- | --- | --- | --- | --- | --- | --- | --- | --- | --- | --- | --- | --- | --- | --- | --- | --- | --- | --- | --- | --- | --- | --- | --- | --- | --- | --- | --- | --- | --- | --- | --- | --- | --- | --- | --- | --- | --- | --- | --- | --- | --- | --- | --- | --- | --- | --- | --- | --- | --- | --- | --- | --- | --- | --- | --- | --- | --- | --- |
