# Supplementary material for: CBS-derived H2S facilitates host colonization of Vibrio cholerae by promoting the iron-dependent catalase activity of KatB
Source: PLoS Pathog. 2021 Jul 20;17(7):e1009763. doi: 10.1371/journal.ppat.1009763 (PMC8324212; doi:10.1371/journal.ppat.1009763)
Supplement: S7 Fig — (PDF) [file ppat.1009763.s007.pdf]

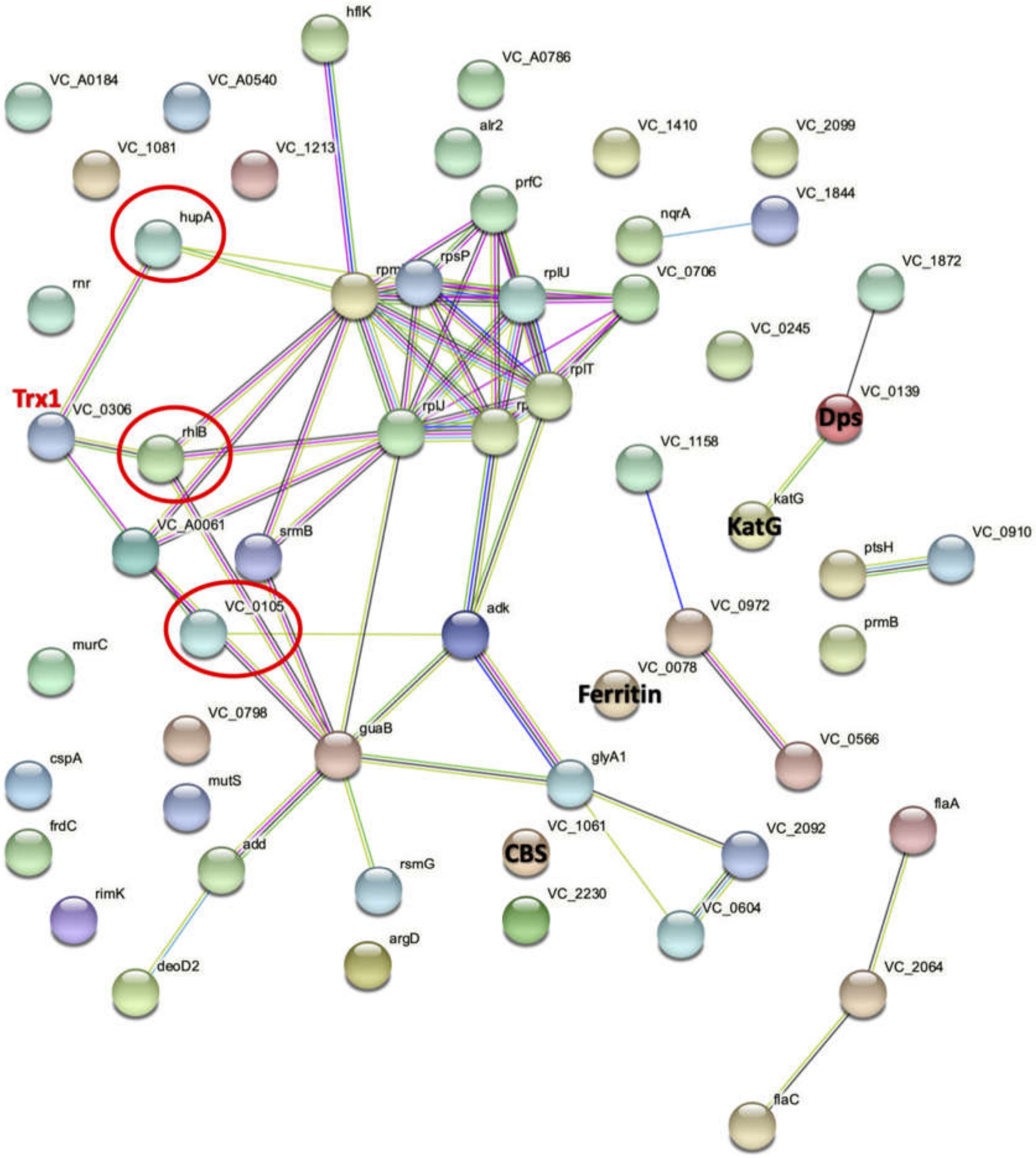

S7 Fig. STRING network analysis of differentially up-regulated proteins in the proteome (S3 Table).
